# Single-cell transcriptome sequencing–based analysis: probing the mechanisms of glycoprotein NMB regulation of epithelial cells involved in silicosis

**DOI:** 10.1186/s12989-023-00543-9

**Published:** 2023-07-19

**Authors:** Shaoqi Yang, Yuheng Sun, Min Long, Xinbei Zhou, Mengqin Yuan, Liliang Yang, Wei Luo, Yusi Cheng, Xinxin Zhang, Wei Jiang, Jie Chao

**Affiliations:** 1 Jiangsu Provincial Key Laboratory of Critical Care Medicine, Department of Physiology, School of Medicine, Zhongda Hospital, Southeast University, 87 Dingjiaqiao Rd, Nanjing, Jiangsu 210009 China; 2 Key Laboratory of Environmental Medicine Engineering, School of Public Health, Ministry of Education, Southeast University, Nanjing, Jiangsu 210009 China; 3 Department of Biomedical Engineering, College of Automation Engineering, Nanjing University of Aeronautics and Astronautics, 29 Jiangjun Avenue, Nanjing, Jiangsu 211106 China; 4 School of Medicine, Xizang Minzu University, Xianyang, Shanxi 712082 China

**Keywords:** Silica, Pulmonary fibrosis, Extracellular matrix, Extracellular vesicles, Epithelial mesenchymal transformation

## Abstract

Chronic exposure to silica can lead to silicosis, one of the most serious occupational lung diseases worldwide, for which there is a lack of effective therapeutic drugs and tools. Epithelial mesenchymal transition plays an important role in several diseases; however, data on the specific mechanisms in silicosis models are scarce. We elucidated the pathogenesis of pulmonary fibrosis via single-cell transcriptome sequencing and constructed an experimental silicosis mouse model to explore the specific molecular mechanisms affecting epithelial mesenchymal transition at the single-cell level. Notably, as silicosis progressed, glycoprotein non-metastatic melanoma protein B (GPNMB) exerted a sustained amplification effect on alveolar type II epithelial cells, inducing epithelial-to-mesenchymal transition by accelerating cell proliferation and migration and increasing mesenchymal markers, ultimately leading to persistent pulmonary pathological changes. GPNMB participates in the epithelial-mesenchymal transition in distant lung epithelial cells by releasing extracellular vesicles to accelerate silicosis. These vesicles are involved in abnormal changes in the composition of the extracellular matrix and collagen structure. Our results suggest that GPNMB is a potential target for fibrosis prevention.

**Supplementary Information:**

The online version contains supplementary material available at 10.1186/s12989-023-00543-9.

## Introduction

Silicon dioxide (SiO_2_) is a widespread environmental toxicant in industrial production [1]. In humans, prolonged inhalation of large amounts of silica can cause persistent pulmonary inflammation, sustained impairment, and eventual progression to irreversible pulmonary fibrosis disease, posing a great threat to human health. The Global Burden of Diseases study described the epidemiological status of silicosis in Asia, with the highest number of global incident cases observed in China (9066 cases) [2]. The lack of effective therapeutic drugs and tools has created an urgent need to explore therapeutic targets for silicosis [3].

Myofibroblasts arise from multiple sources, including resident mesenchymal cells, epithelial cells, endothelial cells, and bone marrow stem cells, and are key cellular mediators of fibrosis [4]. Alveolar epithelial type II cells (AT2) are a heterogeneous population that maintains lung homeostasis in the alveoli in a secretory and regenerative manner [5]. Lung fibroblasts originate during the epithelial–mesenchymal transition (EMT) of alveolar epithelial cells, and their pro-fibrotic phenotype has been shown to contribute to the development of pulmonary fibrosis [5]. Epithelial cells can lose polarity, rearrange their F-actin stress fibers, have enhanced migration capacity [6], promote fibrogenesis [7], and express mesenchymal markers (α-smooth muscle actin [α-SMA] and collagen I), leading to the abnormal accumulation of ECM and scar tissue formation, ultimately resulting in organ dysfunction [8]. Although there is evidence of EMT [9, 10], the specific molecular mechanism still requires in-depth exploration.

Experimental evidence suggests that, in addition to phenotypic alterations, epithelial cells can secrete large numbers of extracellular vesicles (EVs), which carry bioactive molecules, express cell surface proteins similar to their origin, and adhere and fuse to circulating or distant resident cells [11]. Moreover, EVs released from airway epithelial cells may have a regulatory role in the development of pulmonary fibrosis [12].

In this study, a comprehensive analysis based on single-cell RNA sequencing (scRNA-Seq) was performed at the cellular level, and the key molecular glycoprotein non-metastatic melanoma protein B (*Gpnmb*), which precisely regulates EMT, was screened to further explore the underlying mechanism. The *Gpnmb* gene encodes a type I transmembrane glycoprotein [13] and plays a role in regulating cell proliferation, adhesion, differentiation, and extracellular matrix (ECM) protein synthesis [14, 15]. In fibrotic diseases, the exact mechanism of GPNMB involvement in the inflammatory response is unclear; however, it is known to be involved in the progression of fibrosis through upregulation of MMP-3 mRNA expression in mouse fibroblasts, which activates the ERK signaling pathway and induces fibroblast activation and proliferation [16]. This study aims to explore the pathological mechanisms of GPNMB underlying pulmonary fibrosis progression to reveal new therapeutic targets.

## Materials and methods

### Reagents

Silica, 80% of which was less than 5 μm in size and have been characterized [17], was purchased from Sigma® (S5631) and used after drying at 200 °C for 16 h. Silica samples for cellular experiments were suspended in sterile saline (NS) at a concentration of 5 mg/ml, and the dose of silica applied in 24-well plates was 50 μg/cm^2^. Silica suspensions for animal experiments were at a concentration of 50 mg/ml.

### Cell culture

Murine lung epithelial-12 cells and bronchial epithelium transformed with Ad12-SV40 2B (ATCC) were cultured in DMEM supplemented with 10% FBS, 100 U/ml penicillin, 100 μg/ml streptomycin, and 2 mM L-GlutaMAX (obtained from Gibco®) at 37 °C in a humidified 5% CO_2_ atmosphere.

### Silicosis model

Male C57BL/6 mice were purchased from Nanjing Medical University. During the feeding process, the mice were fed and had free access to water, with a temperature controlled at 20–22℃ and light exposure alternating between day and night for 12 h each. All animal procedures were approved by the Laboratory Animal Care and Use Committee of Southeast University (20,210,106,011) and were performed in strict accordance with the National Institutes of Health Guide for the Care and Use of Laboratory Animals. Male C57BL/6 mice were selected at 5–6 weeks of age and anesthetized with 50 mg/kg of 1% sodium pentobarbital solution by intraperitoneal injection. After disinfecting by wiping the neck with 75% alcohol, the subcutaneous tissue was bluntly separated using ophthalmic forceps to expose the trachea. Subsequently, 100 μL of SiO_2_ suspension (50 mg/mL) or an equivalent volume of saline was aspirated with a 1-ml syringe and injected into the trachea after cricoid cartilage puncture. Immediately, the mice were held vertically and the suspension was shaken to achieve a uniform distribution. The mice were divided into NS and SiO_2_ groups according to the modeling reagents.After surgery, the respiration and postoperative activity of the mice were observed to ensure normalcy, before they were sent to the animal room for continued housing until the model was successfully constructed.

### Single-cell transcriptome sequencing

The mouse silicosis model was constructed and lung tissue sampling was performed at 7 and 56 days from one mouse per group. The mouse heart was perfused with pre-cooled PBS buffer at 4°C for body circulation filling, and the perfusion was stopped after the mouse lung tissue became white. Subsequently, the lung tissue was extracted and placed on ice to separate both lung lobes and then washed rapidly with PBS buffer three times. The mouse lung tissues were cut and digested in cell suspensions to ensure 300–600 cells/μL, which were then submitted to CapitalBio Technology Company for scRNA-Seq. Whole lung tissue was cut into approximately 10-mm pieces and dissociated into single cells using a Lung Dissociation Kit (Miltenyi Biotech, 130-095-927, Germany). Using Single-Cell 5’ Library and Gel Bead Kit (10x Genomics, 1,000,169) and Chromium Single-Cell G Chip Kit (10x Genomics, 1,000,120), a cell suspension (300–600 living cells per μl as determined by CountStar) was loaded onto a Chromium single-cell controller (10x Genomics) to generate single-cell gel beads in emulsion (GEMs) according to the manufacturer’s protocol. Fragmented and randomly primed 150-bp paired-end libraries were sequenced using Illumina NovaSeq6000.

### Single-cell transcriptome sequencing analysis

Cell barcode filtering, alignment of reads, and UMI counting were performed with Cell Ranger 6.1.1 (https://www.10xgenomics.com/). Further data analysis was conducted with R v3.2.2 [18] based on the official tutorial in R v3.6.0. A Seurat S4 object for each sample was constructed, and the R package DoubletFinder was used to effectively detect and remove doublets [19]. The doublet rate (the nExp parameter in DoubletFinder) was estimated according to the 10x Chromium User Guide based on the number of recovered cells. Then, the four datasets were combined as one Seurat S4 object. For quality control and filtering, cells with < 500 detected genes or with > 20% mitochondrial gene content were excluded, and genes present in less than 10 cells were deleted. scRNA-seq data were normalized with LogNormalize (scale factor 10,000). Two thousand highly variable genes were identified, and normalized counts were scaled by default. We performed principal component analysis (PCA) for primary dimensionality reduction with 30 dimensions, which was selected based on the elbow plot. Batch effects among four samples were alleviated with Harmony [20]. Thirty-three clusters were identified using the FindNeighbors (based on KNN graphs) and FindClusters (based on Louvain method, resolution = 1) functions in Seurat. Harmony embeddings were used as input for t-Distributed Stochastic Neighbor Embedding (t-SNE) [21] which allows data visualization in a two-dimensional space. Cell type annotation was conducted with the manually curated cell type markers, and 33 clusters were merged into 20 cell types. Pseudotime trajectories determining the transcriptional dynamics among cell types and clusters were constructed using the R package monocle 2 [22, 23]. The expression matrix of selected cells derived from Seurat was used to build a CellDataSet for the Monocle pipeline. The cell trajectory and position with tree structure were plotted, in which the data were reduced to two dimensions through discriminative dimensionality reduction using the DDRTree method, as previous works did in dimension reduction [24–26]. RNA velocity, a high-dimensional vector that predicts the future state of individual cells on a timescale of hours, was used with the R package velocyto.R [27]. We prepared spliced and unspliced expression matrices from files in loom format and performed clustering. Genes were filtered based on the minimum average expression magnitude (in at least one of the clusters). RNA velocity was estimated using a gene-relative model with k = 20 cell kNN pooling and using top/bottom 2% quantiles for gamma fit, and the result was visualized on the Uniform Manifold Approximation and Projection (UMAP) embedding [28].

### Gene expression omnibus(GEO) Data Analysis

Both the GSE24206 and GSE53845 datasets were obtained from the GEO database. Lung tissue samples from patients with idiopathic pulmonary fibrosis (IPF) and healthy controls were used for analysis. In the GSE24206 dataset, samples were collected from 11 patients with IPF (six patients provided a pair of samples from the upper and lower lobes and five patients contributed singleton samples), and six control specimens were obtained from routine lung volume reduction of healthy donor lungs at the time of lung transplantation [29]. In the GSE53845 dataset, RNA was extracted directly from lung tissue samples from 40 IPF patients or eight healthy controls [30].

### Western blotting

The protein levels in MLE-12 cells and EVs were detected by western blotting, and the results were imaged with a Tanon scanner. MLE-12 cells were cultured in 24-well plates, and proteins were extracted with a cell lysis solution (100:1) containing protease inhibitors and placed in a refrigerator at − 80 °C to lyse overnight. Similarly, EV pellets were lysed with lysis buffer. Protein samples were prepared by boiling the samples at 100 °C for 5 min with proteins extracted using a BCA protein quantification kit. The protein samples were separated by sodium dodecyl sulfate-polyacrylamide gel electrophoresis and transferred to PVDF membranes at a constant current of 300 mA. The PVDF membranes were blocked with buffer containing 5% skim milk powder at room temperature for 1 h. Next, the membranes were incubated with the primary antibody for 16 h at 4 °C. The next day, the membranes were washed three times with TBST at room temperature, incubated with the secondary antibody for 1 h at room temperature, and then washed a further three times. Primary antibodies against anti-GPNMB (1:1000, Abcam), anti-GAPDH (1:2000, Proteintech), anti-α-SMA (1:1000, Proteintech), anti-Fn1 (1:1000, Affinity), anti-TSG101 (1:2000, Proteintech), anti-GM130 (1:1000, Proteintech), anti-Alix (1:1000, Proteintech), anti-Calnexin (1:1000, Proteintech), anti-β-actin (1:1000, Proteintech), and anti-Collagen I (1:1000, BioWorld) were used in this study.

### Tissue immunofluorescence staining

Mouse lung tissues were embedded with OCT complex (Sakura, USA), and the lung slices were cut into 8-μm sections and allowed to dry. After washing with PBS to remove the OCT complex, the cells were washed thrice with PBS and incubated with 10% fetal lamb serum in 0.3% Triton X-100 for 2 h at room temperature, before incubating with the primary antibody at 4 °C overnight. The next day, the cells were incubated under light-protected conditions along with a fluorescent dye-coupled secondary antibody (Alexa Fluor, Thermo Fisher Scientific), and the nuclei were stained with 4,6-diamidino-2-phenylindole (DAPI). Tissue sections were observed using a confocal laser scanning microscope (FV3000, Olympus, Japan). Before the experiments, the coverslips were pretreated with polylysine, and then, the cells were inoculated in 24-well plates containing the coverslips. After the experimental treatment, the medium in the 24-well plates was removed, and the cells were washed thrice with PBS and fixed with 4% paraformaldehyde at 4 °C overnight.

### Cell viability

Cells were inoculated in 96-well plates at a density of 5000 cells per well and stimulated with SiO_2_ suspension. After the reaction was completed, CCK-8 reagent (APExBIO, USA) was added at a ratio of 10:1 and co-incubated with the cells for 30 min under light-proof conditions. Finally, the absorbance was measured at 450 nm using a microplate reader (BioTek, USA).

### Phalloidin staining

The cells were incubated until the cell density reached approximately 80%, washed twice with PBS, fixed with 4% paraformaldehyde at room temperature for 10 min, permeabilized with 0.5% Triton X-100 for 5 min, covered with TRITC-labeled Phalloidin staining solution, and incubated at room temperature for 30 min. The cell nuclei were labeled with 4,6-diamino-2-phenylindole (DAPI), if needed. Observation was performed using a confocal laser scanning microscope (FV3000, Olympus, Japan).

### Treatment with tunicamycin

Cells were passaged in 24-well plates and allowed to adhere for 12 h. Tunicamycin was used in pretreatment experiments at a concentration of 5 μg/ml acting for 16 h. GPNMB protein expression was then assessed using western blot analysis [31].

### Preparation of EVs

MLE-12 cells were inoculated in 150 cm^2^ culture dishes (NEST, China) and cultured at 37 °C. After the cell density reached approximately 80%, the SiO_2_ pellet suspension was added to the supernatant and mixed for processing. After 48 h, the supernatant was collected and centrifuged at 300 g for 5 min, 3000 g for 30 min, and 10,000 g for 1 h. The bottom sediment was discarded after each centrifugation. The supernatant was then filtered through a sterile 0.22-μm filter (Millipore, USA) and the pellet was resuspended in PBS at 4 °C using an XPN-100 ultracentrifuge (Beckman Coulter, USA), after which, the resulting pellet was ready for study.

### Examination of EVs using transmission electron microscope (TEM)

The EVs were first immobilized in 4% paraformaldehyde (PFA). Briefly, a drop of EV (10 μL) suspension was air-dried on a formvar carbon-coated electron microscope grid and then stained with 3% phosphotungstic acid (Soleibao Technology, China) for 5 min. Finally, the morphology of EVs was examined using a TEM (Hitachi, Japan).

### Labeling of EVs

EVs were suspended in particle-free phosphate-buffered saline (DPBS, Sigma-Aldrich) at a protein concentration of 100 μg/ml. The resuspended EVs were then stained with Dil dye (Yeasen Biotechnology, China) at 37 °C for 5 min, after which, complete medium was added to terminate the staining reaction. The EV samples were diluted with DPBS and ultracentrifuged at 200,000 g for 2 h at 4 °C. The pellet was gently resuspended in 2 ml DPBS.

### Extracellular matrix preparation

Decellularized ECM was obtained according to a published protocol [32], with some modifications.

### Lentiviral transfection

MLE-12 cells were transfected with the *Gpnmb* overexpression vector (Gpnmb-OE-MLE-12) or negative control vector (*Gpnmb*-NC-MLE-12) at a multiplicity of infection (MOI) of 50 according to the manufacturer’s protocol. The levels of GPNMB in *Gpnmb*-OE-MLE-12 and *Gpnmb*-NC-MLE-12 were estimated via RT-qPCR. Lentiviral transfection was obtained according to a previously published protocol [33], with some modifications.

### Nested matrix model

We used the three-dimensional (3D) migration model described in a previous study with some modifications [34]. For the nested attachment matrix, EVs were transplanted into the ECM and co-incubated for 3 h before adding 200 μL of complete medium per well, after which, epithelial cells were transplanted and incubated with DMEM containing 5% fetal bovine serum in the attached state for 48 h. Subsequently, the ECM was removed from the culture wells and placed in 60 μL of fresh decellularized collagen matrix solution, with the solution centered on the scratched area (10-mm diameter). The newly transferred epithelial cell-filled collagen matrix was then covered with 140 μL of decellularized collagen matrix solution. After incubation for 1 h, 800-μL DMEM containing 10% FBS was added to the wells. Cell migration at 12, 24, and 48 h was captured and digitally imaged using an EVOSFL cell imaging microscope (Thermo Fisher Scientific) and compared with that observed at 0 h.

### Statistical analysis

Statistical analyses were performed using GraphPad Prism 8.0.2 software (GraphPad Software, USA). Two-group comparisons were assessed for significance using a two-tailed Student’s *t*-test. Multiple group (three or more groups) comparisons were performed using one/two-way ANOVA. All data are expressed as the mean ± SEM, and differences were considered statistically significant when *P* < 0.05.

## Results

### Analysis of alveolar type II epithelial cell fate changes by single-cell transcriptome sequencing

**Fig. 1:**
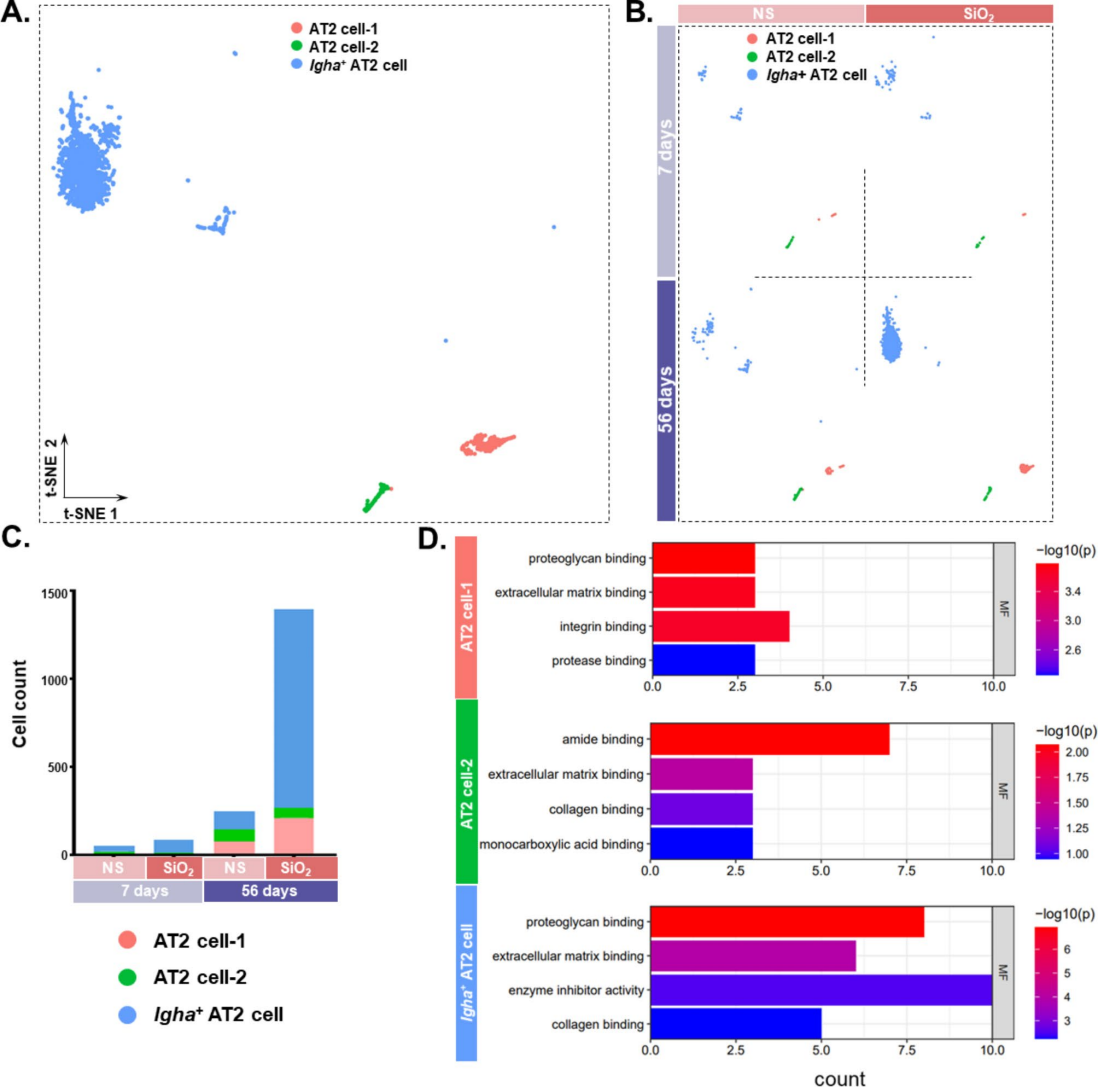
Probing alveolar type II epithelial cell fate changes via single-cell transcriptome sequencing. **A**. t-SNE demonstrating three subtypes of AT2. **B**. t-SNE demonstrating each subtype of AT2 in the saline and silica groups at 7 and 56 days. **C**. Changes in the number of alveolar type II epithelial cells. **D**. GO enrichment analysis of the differential genes (*P < 0.05, avg_logFC > 0) for each subtype of AT2 in the saline and silica groups at 7 and 56 days

**Fig. 2:**
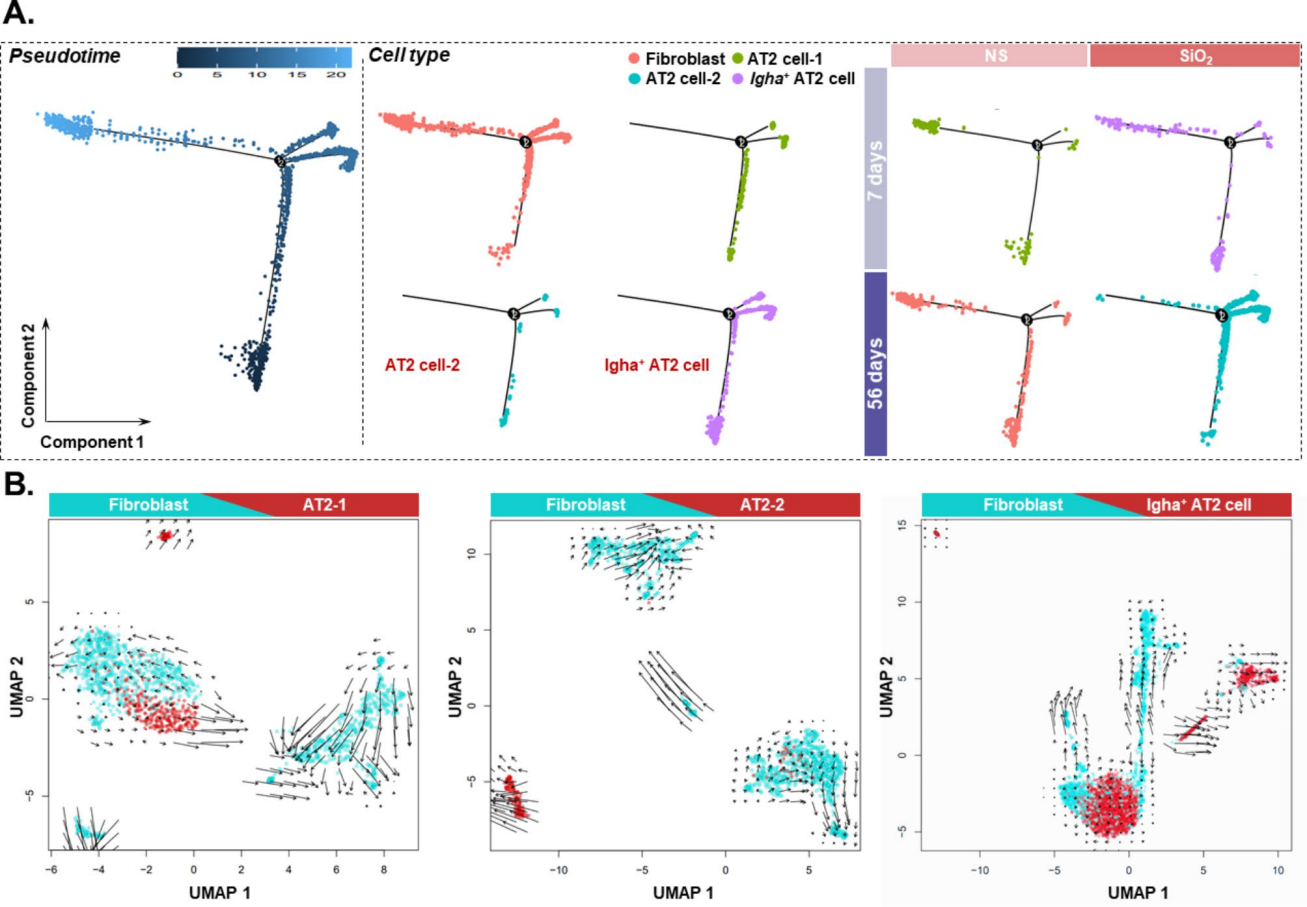
Probing alveolar type II epithelial cell transition via bioinformatics analysis. **A**. Pseudo-time-series analysis showing the differentiation trajectory of AT2 and fibroblasts. **B**. RNA velocity analysis of fibroblasts and three cell types of AT2.

To assess the changes in lung tissue cell composition after SiO_2_ exposure, we performed scRNA-Seq on lung tissue samples. A total of 20 cell type were identified and viewed in two dimensions using t-distribution random neighborhood embedding (t-SNE) (Fig. S1A). To investigate the role of alveolar type II epithelial cells in the progression of silicosis, a silicosis model was first established in mice by tracheal drip of SiO_2_, and a simultaneous tracheal drip of saline was set as the control group. Finally, whole lung tissue samples were obtained from four groups (NS-7-day group, SiO_2_-7-day group, NS-56-day group, and SiO_2_-56-day group), and single cell transcriptome sequencing analysis was performed. The alveolar type II epithelial cells could be classified into three subtypes: AT2-1, AT2-2, and *Igha*^+^ AT2 (Fig. 1A and Fig. S1B), and significant changes in cell numbers were observed in all four groups (Fig. 1B), with the highest number of cells observed in the SiO_2_-56d group (Fig. 1C). Gene Ontology enrichment analysis was performed after screening for differentially expressed genes between groups of SiO_2_-56d and NS-56d, and the results suggested molecular functions of ECM binding and collagen binding (Fig. 1D). Next, the origin and purpose of AT2 and lung fibroblasts were analyzed and explored by pseudo-time-series analysis. The results showed that all three subtypes of AT2 have a tendency to undergo a transition to fibroblasts (Fig. 2A). To further validate the results, RNA velocity analysis was performed on the three subtypes of alveolar type II epithelial cells and lung fibroblasts. The results suggested differentiation in all three subtypes (Fig. 2B).

### Epithelial cells likely undergo EMT

**Fig. 3:**
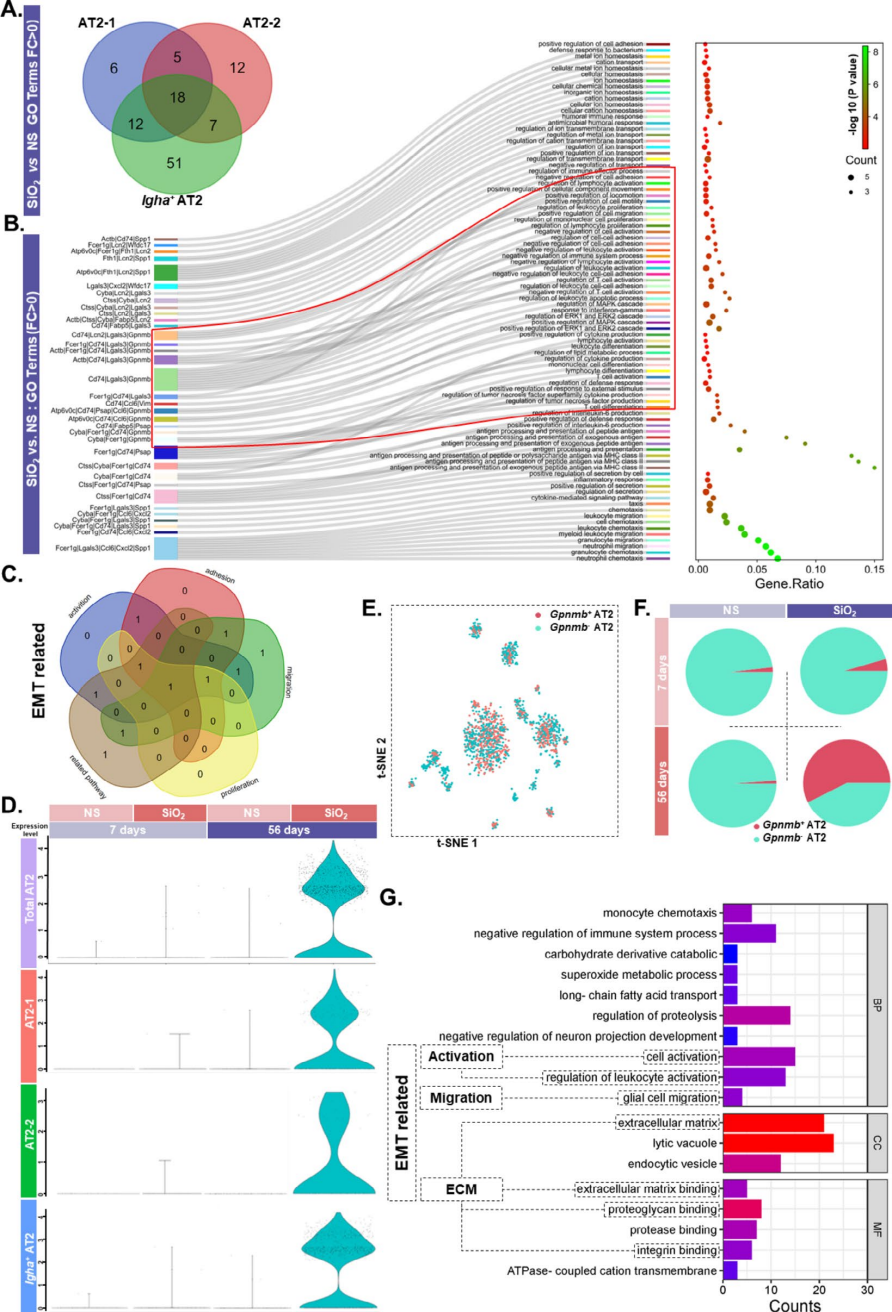
Screening of key molecules involved in the occurrence of EMT phenomenon in alveolar II epithelial cells. **A**. Venn diagram showing the differential genes for the three subtypes of AT2 (*P < 0.05, avg_logFC > 1.0). **B**. Sankey diagram showing the function of 18 differential genes. **C**. Venn diagram showing the differential genes for the three subtypes of AT2. **D**. Violin plot showing the expression of *Gpnmb* in AT2. **E**. t-SNE plot showing the distribution of the two subtypes of AT2, *Gpnmb*^*+*^*AT2*, and *Gpnmb*^*−*^*AT2.***F**. Pie chart showing the percentage of each AT2 subtype occupying the total number of AT2 cells. **G**. GO enrichment analysis of the top 20 differential genes

To investigate the genes that play a key role in epithelial mesenchymal transition, we screened the three subtypes for differentially expressed genes (avg_logFC > 0, *P* < 0.05) among the 56-day groups and extracted the intersections, which resulted in a total of 18 identical genes being screened (Fig. 3A). A Sankey diagram shows the results of GO enrichment analysis, demonstrating that the entries were enriched with EMT-related functions (e.g., cell migration, cell adhesion, and cell proliferation), most of which are associated with *Gpnmb* (Fig. 3B). The enrichment analysis results suggest that *Gpnmb* is involved in regulating the migration, activation, proliferation, adhesion, and EMT-related signaling pathways of the EMT phenotype (Fig. 3C). Next, bioinformatics analysis was used to process the data to determine whether *Gpnmb* is involved in regulating EMT. The results revealed high expression of *Gpnmb* in SiO_2_-56d and no significant elevated expression in SiO_2_-7d, as shown in the violin plot (Fig. 3D), which is consistent with the expected hypothesis. All alveolar type II epithelial cells were divided into two groups, i.e., as *Gpnmb*^*+*^AT2 and *Gpnmb*^−^AT2, respectively (Fig. 3E). The results showed that *Gpnmb*^*+*^ epithelial cells appeared increased in number at SiO_2_-56d (Fig. 3F), where the enrichment analysis of the two isoforms differentially expressed gene (avg_logFC > 0, *P* < 0.05) indicated an association with cell migration and activation, as well as the ECM (Fig. 3G). The expression of mesenchymal cell marker (N-Cad) was elevated in the silicosis model, and co-localization with epithelial cell marker (E-Cad) occurred (Fig. S1C). The ratio of E-Cad to N-Cad about NS-56d is 1.577 and SiO_2_-56d is 1.140.

### GPNMB mediates the SiO_2_-induced proliferation and activation of MLE-12 cells

**Fig. 4:**
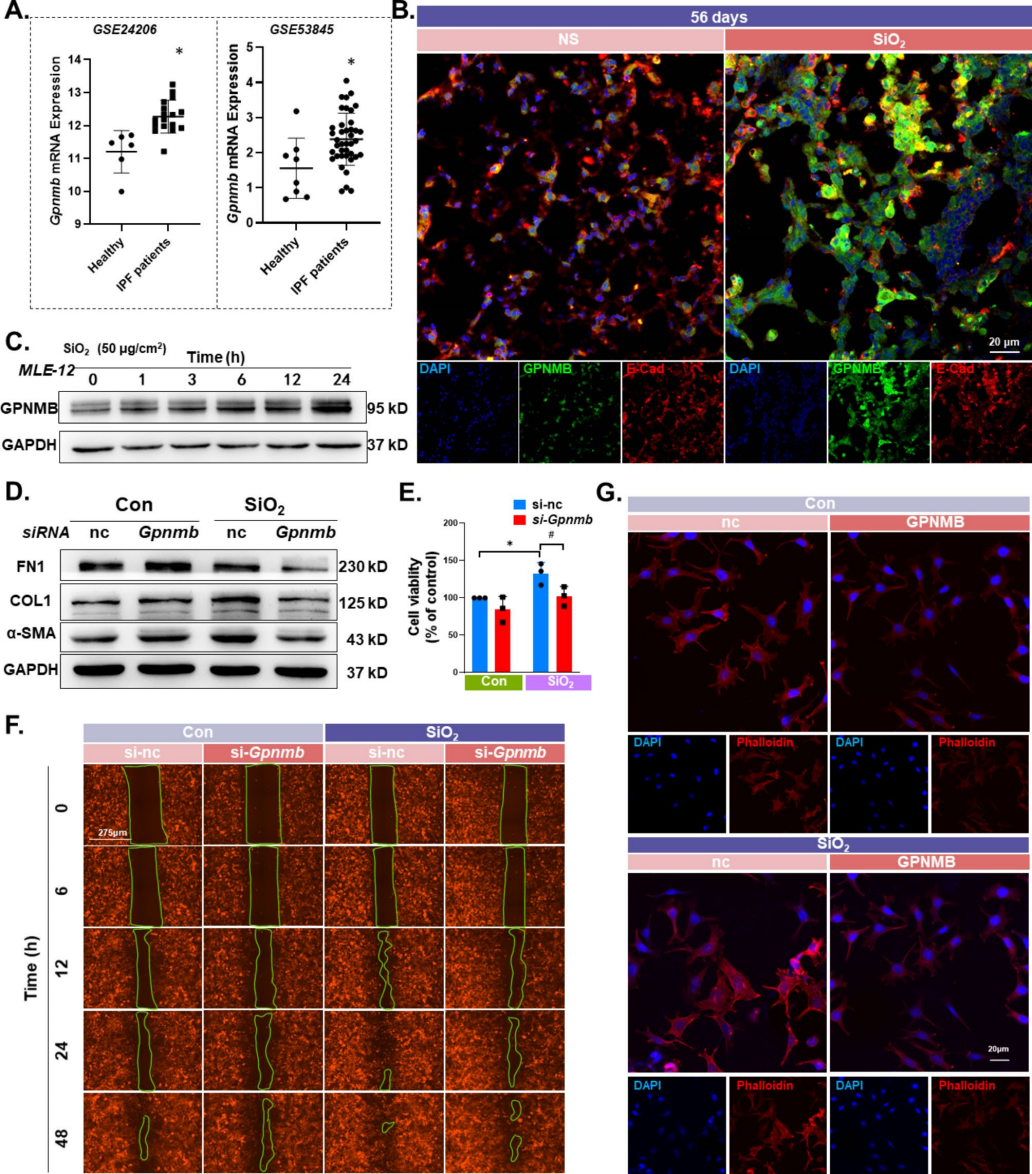
GPNMB participation in the EMT process. **A**. Expression of GPNMB in the lung tissue of patients with IPF was significantly higher than that of the healthy group in the GEO database (*P < 0.05). **B**. Immunohistochemical staining showing that GPNMB was expressed on epithelial cells. Expression was higher in the experimental group than in the control group, scale bar: 20 μm. **C**. Representative western blot results showing that the expression of GPNMB was elevated over time in MLE-12 cells. **D**. Representative western blot results showing that the downregulation of GPNMB partially reversed the SiO_2_-induced upregulation of FN1, COL1, and α-SMA. **E**. CCK-8 assay results: #*P* < 0.05 indicates that the *si-Gpnmb* group had lower cell viability levels after SiO_2_ treatment than the *si-nc* group. *P < 0.05 indicates a significant increase in the viability of MLE-12 cells after 24 h of SiO_2_ treatment compared to the Con-nc group. **F**. Wound-healing experiments showing that downregulation of *Gpnmb* expression attenuated SiO_2_-induced cell migration. **G**. Phalloidin assay showing that the cytoskeleton distribution of microfilaments was coarser in the experimental group than in the control group, and the cytoskeleton level in the *si-Gpnmb* group was lower than that in the *si-nc* group, scale bar: 20 μm

Migration, activation, and proliferation are important phenotypic changes in EMT. To investigate the inevitability and consistency of elevated GPNMB expression in the silicosis model, we verified this phenomenon from different perspectives. The changes in *Gpnmb* mRNA expression levels during human idiopathic pulmonary fibrosis (IPF) were analyzed using the GEO databases. The results showed that *Gpnmb* expression was significantly higher in the IPF patient group than in the healthy group (Fig. 4A), suggesting a role of *Gpnmb* in pulmonary fibrosis. The authenticity and reliability of the results were verified by histochemical staining of mouse lung tissue sections, which showed that GPNMB was expressed at high levels in the SiO_2_-56 d model group and co-localized with the epithelial cell marker (E-Cad) (Fig. 4B). Moreover, a significant increase in the expression level of GPNMB after SiO_2_ stimulation of epithelial cells was verified by western blot, in an in vitro model of the MLE-12 cell line (Fig. 4C and Fig. S2A) and in an in vitro model of human lung epithelial cells (Fig. S2B). To demonstrate that *Gpnmb* is central to the epithelial mesenchymal transition process, subsequent analysis of the protein interactions was conducted (Fig. S2C), the results of which suggested that GPNMB interacts with mesenchymal marker proteins. Migration, proliferation, and activation of alveolar type II epithelial cells are important phenotypes of EMT. Therefore, we investigated the effect of GPNMB on the function of EMT in MLE-12 cells. siRNA knockdown of *Gpnmb* expression was applied to verify whether GPNMB regulates EMT in AT2 (Fig. S2D). Specific downregulation of *Gpnmb* partially attenuated the increase in FN1, COL1, and α-SMA expression induced by SiO_2_ stimulation (Fig. 4D and Fig. S2E). The Cell Counting Kit-8 assay results also showed that GPNMB partially reversed the increase in cell viability (Fig. 4E), and cell migration was suppressed when GPNMB expression was reduced (Fig. 4F and Fig. S2F). Phalloidin staining experiments demonstrated that the microfilaments of MLE-12 cells were thickened by SiO_2_ treatment, and the phenomenon was significantly reversed by *Gpnmb* knockdown (Fig. 4G).

### Glycosylation level of GPNMB regulates the function of EMT

**Fig. 5:**
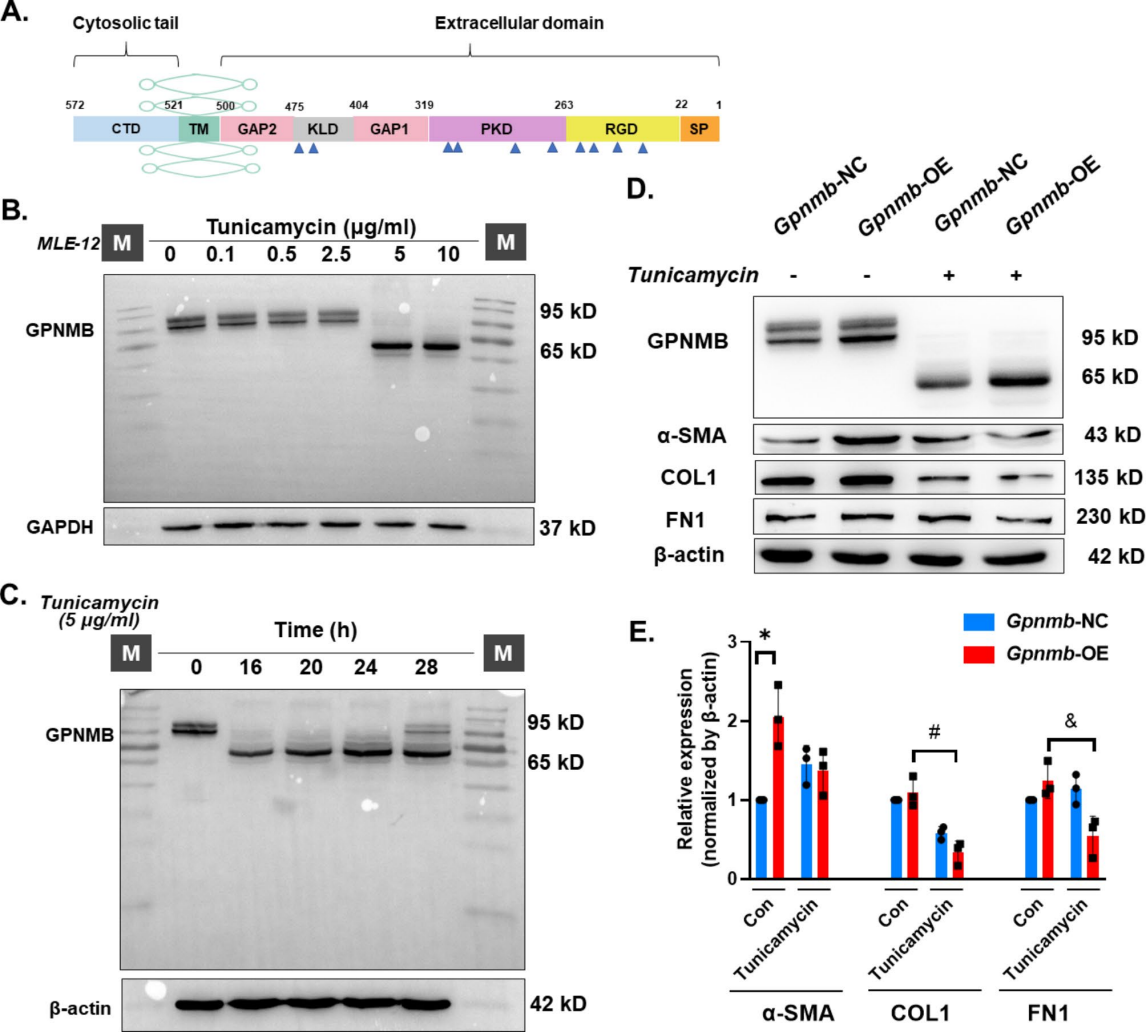
Glycosylation level affects*Gpnmb* function. **A**. Structural domain of GPNMB protein. **B**. Molecular weight of GPNMB changed from 95 kD to 65 kD following treatment with 5 μg/ml of tunicamycin. **C**. Representative western blot results showing an action time of 16 h following treatment with 5 μg/ml of tunicamycin. **D**. Representative western blot results showing that the *Gpnmb*-OE group causes a reversal of the tunicamycin-induced elevation in α-SMA, COL1, and FN1 proteins compared to the *Gpnmb*-NC group. **E**. *P < 0.05 indicates that the α-SMA protein levels were higher in the *Gpnmb*-OE group than in the control group; #*P* < 0.05 indicates that the COL1 expression levels were lower in the *Gpnmb*-OE group than in the control group after tunicamycin treatment; and &*P* < 0.05 indicates that FN1 expression levels were lower in the *Gpnmb*-OE group than in the control group after tunicamycin treatment

A literature search revealed that the molecular function of GPNMB, a transmembrane type I glycoprotein, is related to its degree of glycosylation, with 11 glycosylation sites predicted through the UniProt website (Fig. 5A and Fig. S3A) [31, 35]. Glycosylation was blocked by the N-glycosylation pharmacological inhibitor tunicamycin. After 16 h of action at an optimal concentration of 5 μg/ml (Fig. 5B–C), a significant reduction in the degree of GPNMB glycosylation was observed and the molecular weight of GPNMB decreased from 115 kDa to 65 kDa. Using lentiviral constructs of *Gpnmb-OE* cell lines (Fig. S3B), it was found that high expression of GPNMB could upregulate the expression of FN1, COL1, and α-SMA, which could be reversed by decreasing the level of GPNMB glycosylation (Fig. 5D–E). Therefore, we hypothesized that the different levels of GPNMB glycosylation affected its ability to perform EMT functions.

### GPNMB released into the extracellular compartment via EVs is implicated in the formation of the fibrotic microenvironment

**Fig. 6:**
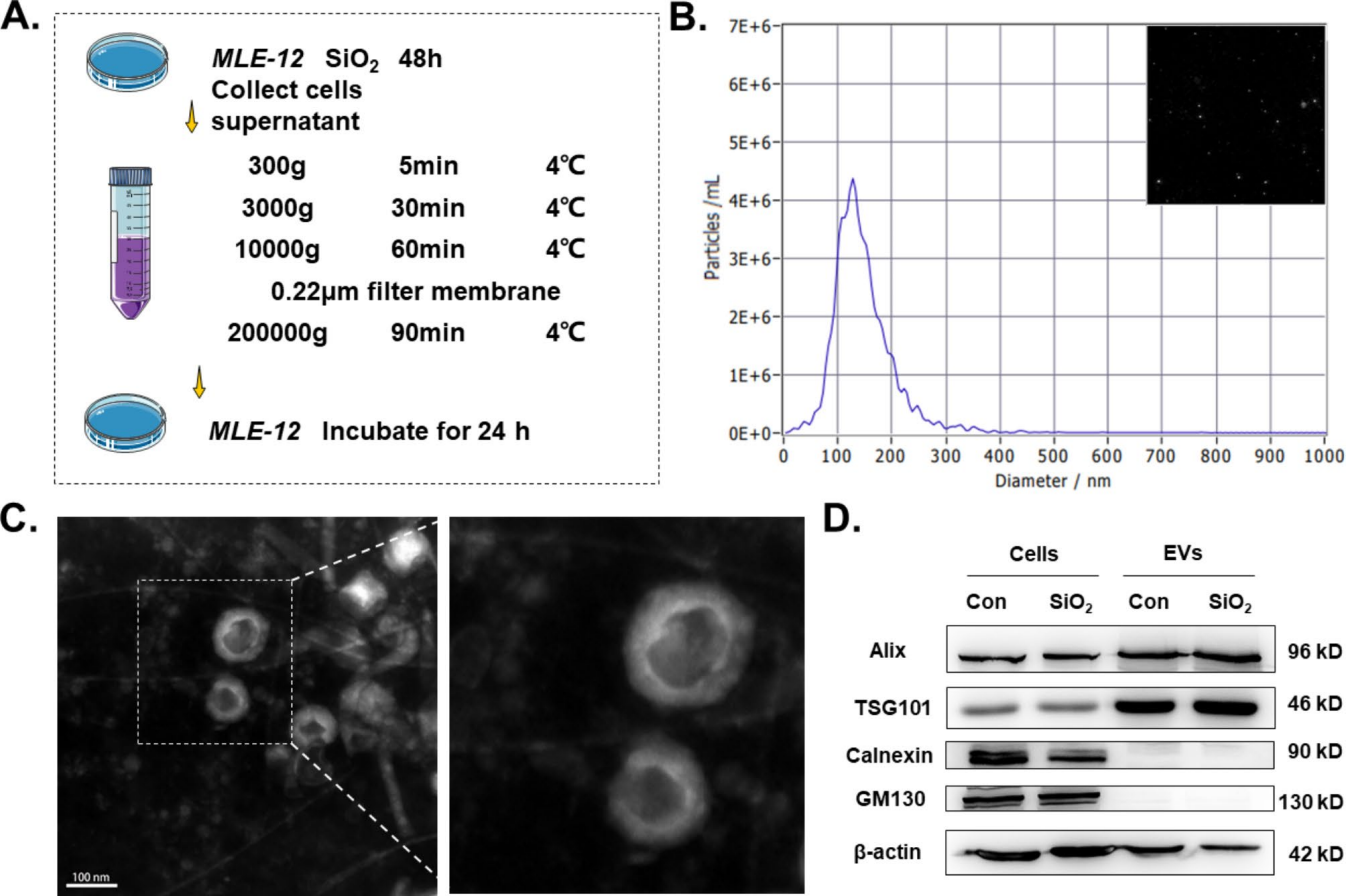
Stimulation by SiO_2_ induces elevated GPNMB expression in EVs released from epithelial cells. **A**. Schematic diagram of the acquisition of EVs from MLE-12 cells. **B**. Measurement of EVs with NTA. **C**. Transmission electron microscopy of EVs isolated from MLE-12 cell cultures. **D**. Western blot analysis of Alix, TSG101, Calnexin, and GM130 in MLE-12 cells and MLE-12-EVs.

GPNMB was released into EVs to further regulate cell proliferation and differentiation [36], and GO enrichment analysis suggested that *Gpnmb*^+^AT2 was associated with EVs. Next, we sought to assess the effect of GPNMB in EVs on epithelial cell function. Following SiO_2_ stimulation of MLE-12 cells for 48 h, EVs were collected by gradient centrifugation (Fig. 6A). NTA determined that the diameter of MLE-12 EVs peaked at 126.7 nm (Fig. 6B), with a saucer-like structure (Fig. 6C). Protein-level analysis further confirmed the properties of MLE-12 EVs (Fig. 6D), confirming the accuracy of the experimentally obtained EVs.

### EVs encapsulate GPNMB to exert fibrotic functions

**Fig. 7:**
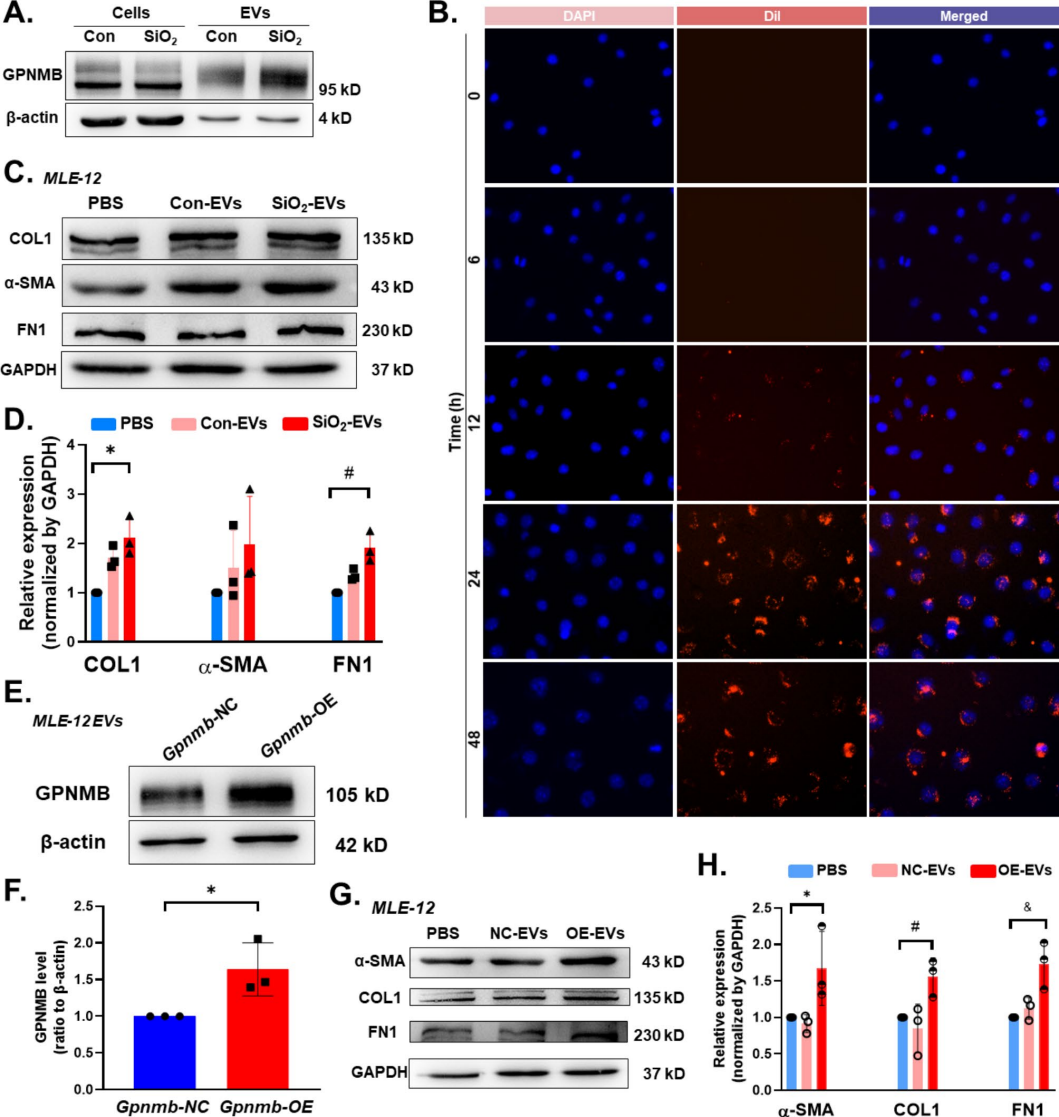
MLE-12 endocytosis of highly expressed *Gpnmb* EVs promotes the EMT process. **A**. Representative western blot results showing higher GPNMB expression in EVs in the SiO_2_ group than in the control group. **B**. Images of EVs incubated with MLE-12 for 0, 6, 12, 24, and 48 h. **C**. Representative western blot results showing elevated protein levels of COL1, FN1, and α-SMA in the SiO_2_-EVs group compared to the control group. **D**. *P < 0.05 indicates higher COL1 protein levels in the SiO_2_-EVs group compared to the control group, while #*P* < 0.05 indicates higher FN1 protein levels in the SiO_2_-EV group compared to the control group. **E**. Representative western blot results showing elevated expression of GPNMB in EVs secreted by cells in the *Gpnmb*-OE group compared to the *Gpnmb*-NC group. **F**. *P < 0.05 indicates that the GPNMB protein levels were higher in the *Gpnmb*-OE group than in the *Gpnmb*-NC group. **G**. Representative western blot results showing elevated protein levels of COL1, FN1, and α-SMA in the *Gpnmb*-OE group compared to the control group. **H**. *P < 0.05 indicates that the α-SMA protein level was higher in the *Gpnmb*-OE group compared to the control group; #*P* < 0.05 indicates that the COL1 protein level was higher in the *Gpnmb*-OE group compared to the control group; and &*P* < 0.05 indicates that the FN1 protein level was higher in the *Gpnmb*-OE group compared to the control group

To investigate whether the function of EVs released from epithelial cells is regulated by the involvement of GPNMB, the obtained EVs were stimulated with SiO_2_. The molecular weights of GPNMB in MLE-12 cells were 95 kDa and 115 kDa, and the highly glycosylated form of 115 kDa can be released into the extracellular compartment via EVs (Fig. 7A and Fig. S4. Next, a live cell imaging system was used to observe the dynamic uptake process of MLE-12 EVs at different time points in vitro. The results showed that Dil-labeled MLE-12 EVs (red fluorescence) gradually increased with increasing co-incubation time (Fig. 7B). The release of SiO_2_-EVs into the extracellular compartment consistently increased the protein levels of the mesenchymal markers COL1 and FN1 (Fig. 7C–D). High expression of GPNMB at the cellular level could promote elevated expression of GPNMB in exosomes released from cells (Fig. 7E–F). Moreover, EVs released from *Gpnmb-OE* cells (OE-EVs) could promote the expression of the mesenchymal markers α-SMA, COL1, and FN1 (Fig. 7G–H).

### GPNMB accumulates on ECM as an exosome carrier with observable function

**Fig. 8:**
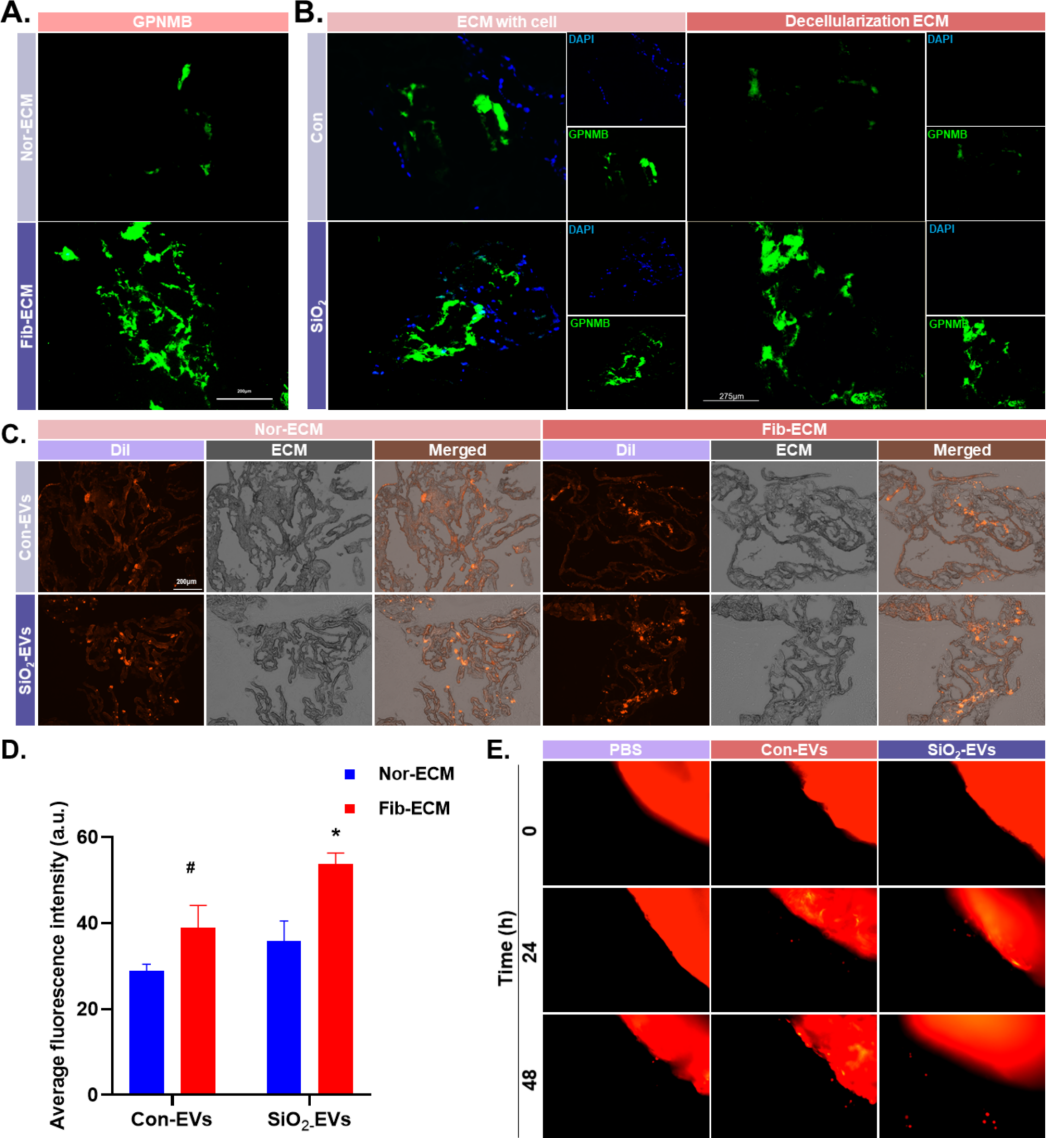
Secreted MLE-12 EVs adhere to the extracellular matrix to exert their effects. **A**. Immunofluorescence staining to assess the GPNMB protein levels in the fibrotic ECM, scale bar: 200 μm. **B**. Elevated GPNMB protein levels in the fibrotic ECM originate from alveolar type II epithelial cells, scale bar: 275 μm. **C**. Adhesion of Dil-labeled EVs to the ECM, scale bar: 275 μm. **D**. Increased adhesion of EVs to Fib-ECM compared to Con-ECM. **E**. Three-dimensional cell migration assay showing that SiO_2_-EVs migrated from Fib-ECM at 24 h quicker than the control

ECM proteomics techniques were used to study the changes in silicosis ECM more specifically. The histological results suggested that GPNMB was the most upregulated protein in fibrotic ECM compared to normal ECM (Fig. S5A). This was verified using ECM tissue immunofluorescence (Fig. 8A), which demonstrated that fibrotic ECM has more GPNMB protein accumulation than normal ECM. Transplantation of MLE-12 demonstrated that the GPNMB protein accumulated on fibrotic ECM was partially secreted by alveolar type II epithelial cells (Fig. 8B). MLE-12 EVs could adhere to the ECM, and Dil-labeled EVs showed higher fluorescence intensity on the fibrotic ECM, indicating stronger adhesion of fibrotic ECM to EVs (Fig. 8C–D). Compared with normal ECM, SiO_2_-EVs could carry more GPNMB protein on fibrotic ECM and played a role in promoting the migration of MLE-12 cells (Fig. 8E and Fig. S5B–C).

## Discussion

Silicosis, caused by continuous inhalation of environmental silica dust, is a major public health problem worldwide. As silicosis currently has no effective drugs or treatments [3], the exploration of therapeutic targets is urgently required. Repeated exposure to silica induces airway epithelial cell damage [37]. Epithelial cells participate in the fibrotic process through EMT, acquiring some mesenchymal features that promote collagen accumulation and ECM migration and production in the lung. EMT leads to the production of the fibrosis marker proteins FN1 [38], COL1, and A-SMA by AT2 [39, 40], which affect cell proliferation and migration. COL1 and FN1 are important components of the ECM [39], and excessive accumulation of ECM can impair pulmonary function.

Studies have demonstrated that EMT is an important factor in the development of pulmonary fibrosis, while inhibition of EMT with TGF-β inhibitors adequately ameliorates pulmonary fibrosis in mice [41]. TGFβ1 activates several non-SMAD signaling pathways, including mitogen-activated protein kinase (MAPK) and AKT, which activates the EMT process. Compared to traditional studies on signaling pathways related to fibrogenic factors, we identified the key genes involved in regulating EMT and further explored their detailed mechanisms of action based on a comprehensive analysis of scRNA-Seq of silicosis using a mouse model of silicosis with airway silica perfusion.

After fractionation of alveolar type II epithelial cells, bioinformatics analysis suggested data support for EMT, while simultaneous experimental data confirmed the development of this phenomenon. GPNMB was identified as a key gene regulating the involvement of alveolar type II epithelial cells in EMT and was originally identified as a type I transmembrane glycoprotein in a hypermetastatic melanoma cell line [42]. *Gpnmb* plays a role in osteogenesis, regulation of proliferation, migration, and differentiation of cells, inflammatory responses [43], and EMT [44, 45]; however, the mechanisms underlying the pathological changes it provokes remained unclear. In this study, we attempted to reveal the full mechanism of GPNMB involvement in the pulmonary fibrosis process using multiple modalities.

In an early pathological model of tracheal silica perfusion, we demonstrated that GPNMB is expressed in AT2 and regulates the cells to undergo phenotypic alterations. As GPNMB is a highly glycosylated protein, some studies have focused on whether the glycan on GPNMB itself regulates its own function [35, 46]. There are two isoforms of GPNMB in AT2: the glycosylated isoform at 90 kDa and the highly glycosylated/matured isoform at 115 kDa, with the 115 kDa glycosylated/matured isoform being the secreted isoform of the protein [46]. Tunicamycin, as an N-glycosylation inhibitor, inhibits the level of GPNMB glycosylation, resulting in the intracellular GPNMB protein form in the immature isoform of 65 kDa, and inhibits the extracellular N-glycosylation of GPNMB such that the fibrogenic function is inhibited to some extent. Although we did not establish the specific glycosylation sites that affect the fibrogenic effect of GPNMB, the experimental results suggest that altered glycosylation levels of GPNMB affect its EMT function to some extent, suggesting that GPNMB represents a novel therapeutic target for pulmonary fibrosis.

Many studies have investigated the role of EVs in pathological processes, such as cancer and immune diseases, as well as in a diverse range of biological functions, such as ECM production and remodeling. As AT2 damage increases, GPNMB proteins are enriched to a certain extent in the cell membrane and secreted extracellularly via vesicles.

Our study showed that the molecular weight of GPNMB protein in vesicles of AT2 was 115 kDa and no other forms were detected; therefore, the highly glycosylated/matured isoform of GPNMB could be secreted to mediate extracellular effects in the form of EVs. Recent studies have shown that EVs carry molecules such as mRNA and proteins to other cells, which leads to changes in the gene expression and phenotype of recipient cells [47]. EVs serve as important mediators of cell migration. EVs released from cancer-related fibroblasts (CAF) have been shown to promote the migration or metastasis of cancer cells by delivering various cargoes, including miRNA and proteins [48]. Here, with increasing fibrotic pathology, EVs carrying GPNMB proteins were released extracellularly and internalized by normal cells not directly stimulated by SiO_2_ to receive indirect signaling stimuli, resulting in a cascade response with amplified fibrogenic effects. Ultimately, epithelial-derived EVs adhere to the distant ECM via humoral circulation, participating in the formation of the fibrotic microenvironment and promoting phenotypic changes in the cells surrounding the ECM.

The ECM, as an important component of tissue, not only provides an important physical scaffold for resident cells in the lung but is also critical for lung cell signaling. Lung cells regulate the production and deposition of ECM, and a positive feedback loop between cells and the ECM induces a constitutive shift in ECM proteins [40], where changes in the collagen structure alter the cell morphology and migration rate [49]. Recent studies have shown that EVs persistently circulate with body fluids to the distal lung tissue and anchor to ECM components via adhesion molecules, resulting in a fibrosis-like microenvironment. In this study, ECM collagen remodeling and component changes were found to recruit more EVs, indirectly changing the ECM composition and acting as a positive regulator. EVs that adhered to the ECM could still be endocytosed by the adjacent cells, and we hypothesize that the fibrotic ECM could play a more temporary role in hiding and accumulating EVs due to the altered collagen structure [50].

It has been shown that EVs can bind to ECM components in the form of adhesion molecules, such as intercellular adhesion molecule-1 (ICAM-1) and the integrin family [51], although the exact mode of adhesion is not discussed in this paper and will be investigated in the future. We hypothesize that EVs can be used as biomarkers of fibrosis in disease states where dynamic remodeling of the ECM occurs.

This study uses the timeline as the main line of disease research to explore the pathological mechanisms of GPNMB involvement in functional alterations of AT2 from multiple perspectives. This explains that the persistent deterioration observed in silicosis is partly due to the extensive pathway of action of the causative gene and the amplification effect of the cascade reaction, which further creates a fibrotic microenvironment in which normal cells are also altered, leading to irreversible disease outcome.

## Conclusion

**Fig. 9:**
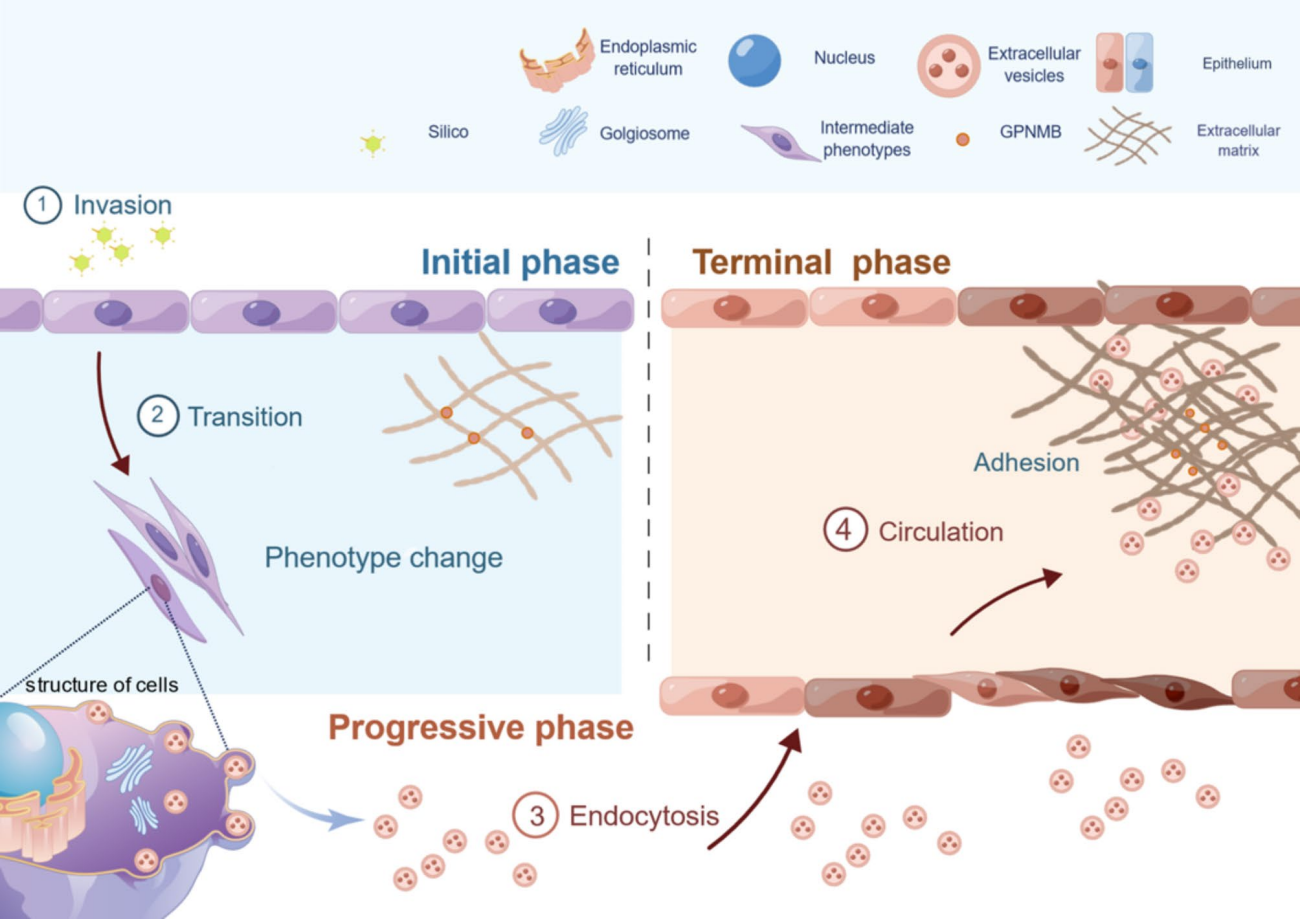
Schematic representation of the mechanism of GPNMB regulation of silica-induced pulmonary fibrosis in alveolar type II epithelial cells. GPNMB expression in AT2 exposed to SiO_2_ was increased, promoting cell proliferation, accelerating migration, and regulating the EMT process. GPNMB was persistently highly expressed in AT2 and can be released extracellularly with extracellular vesicles to exert its effects, participating in the regulation of phenotypic transformation and functional changes in normal AT2. Fibrotic ECM showed abnormal accumulation of GPNMB, part of which originated from AT2, and GPNMB adhered to ECM in the form of extracellular vesicles to participate in the pulmonary fibrosis process and continued to exert a sustained amplifying effect on ECM accumulation

In conclusion, our novel results suggest that alveolar type II epithelial cells stimulated by SiO_2_ activate EMT by upregulating *Gpnmb* and its subsequent release into the extracellular space in the form of vesicles to exert this effect continuously. Notably, alveolar type II epithelial cells sustained EMT by wrapping GPNMB protein in EVs and releasing it into the extracellular space. Subsequently, GPNMB accumulates in the ECM, promoting collagen deposition and ECM component changes. In addition, EVs adhering to the ECM can still be endocytosed by adjacent cells, promoting changes in cellular function (Fig. 9). Therefore, *Gpnmb* represents a critical therapeutic target for silicosis, and these findings will contribute to a better understanding of the mechanisms regulating pulmonary fibrosis progression and pulmonary fibrosis.

## Electronic supplementary material

Below is the link to the electronic supplementary material.

Supplementary Material 1

Supplementary Material 2

## Abbreviations

BCA: BCA Protein Assay Kit
BEAS-2B: Bronchial epithelium transformed with Ad12-SV40 2B
DMEM: Dulbecco’s Modification of Eagle’s Medium
ECM: Extracellular matrix
EVs: Extracellular vesicles
FBS: Fetal bovine serum
MLE-12: Mouse lung epithelial-12 cells
NS: Normal saline
OCT: Optimum cutting temperature compound
PBS: Phosphate-buffered saline
PVDF: Polyvinylidene fluoride
SiO_2_: Silicon dioxide
