# Supplementary material for: Single-cell transcriptome sequencing–based analysis: probing the mechanisms of glycoprotein NMB regulation of epithelial cells involved in silicosis
Source: Part Fibre Toxicol. 2023 Jul 19;20:29. doi: 10.1186/s12989-023-00543-9 (PMC10354944; doi:10.1186/s12989-023-00543-9)
Supplement: Supplementary file 1 — Supplementary Material 1 [file 12989_2023_543_MOESM1_ESM.pptx]

## Slide 1
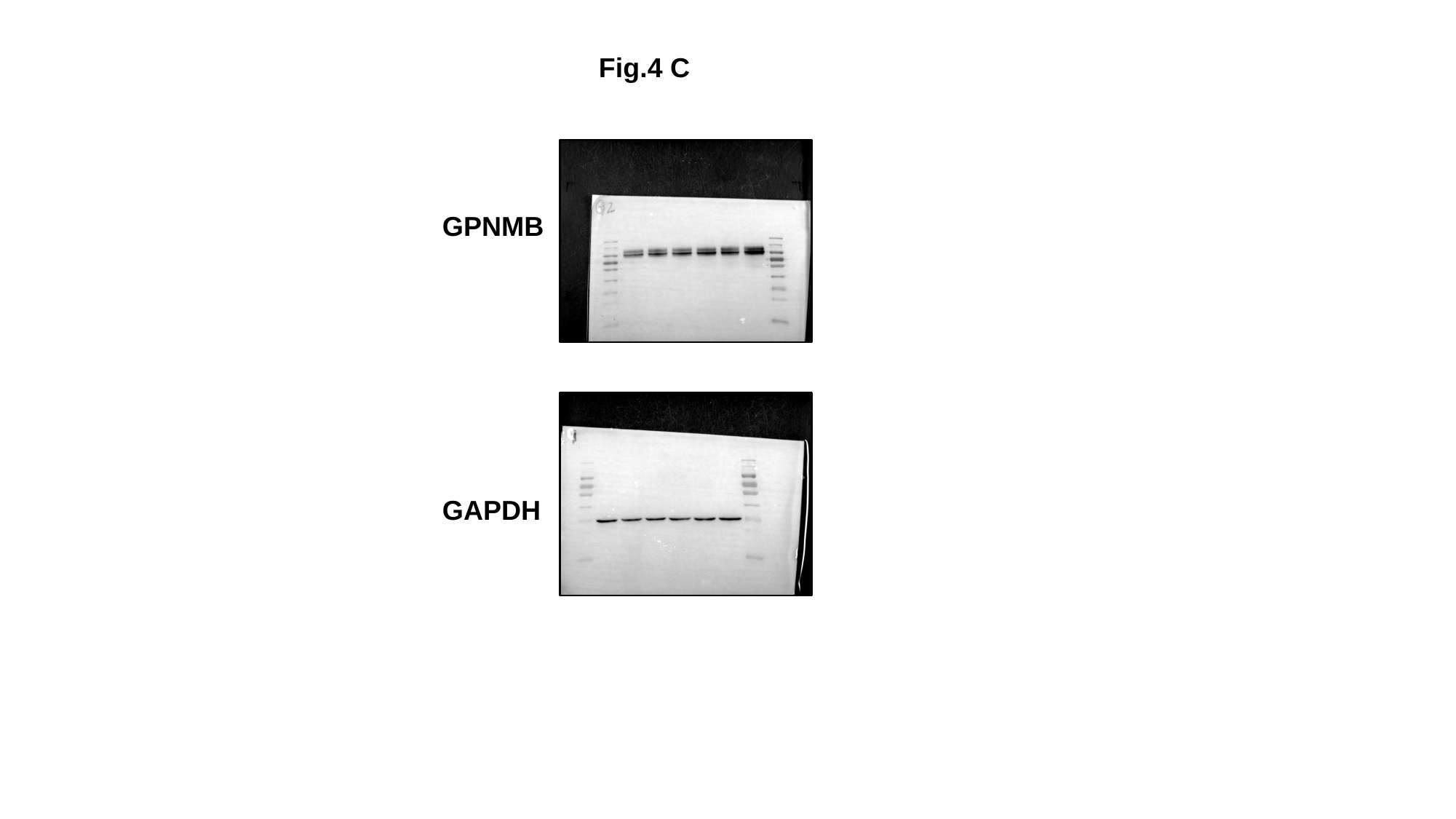

Fig.4 C
GPNMB
GAPDH

## Slide 2
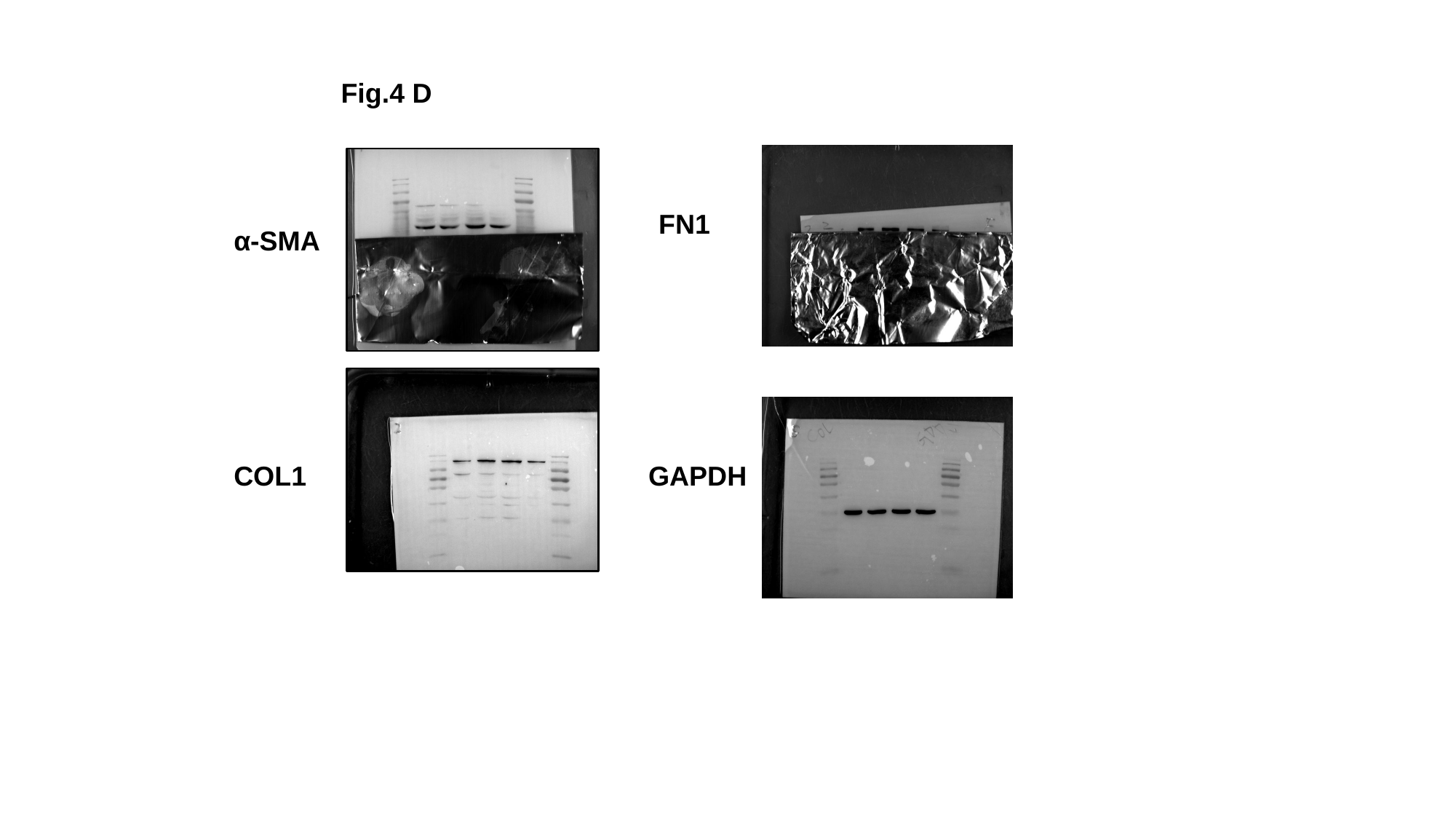

Fig.4 D
FN1
α-SMA
COL1
GAPDH

## Slide 3
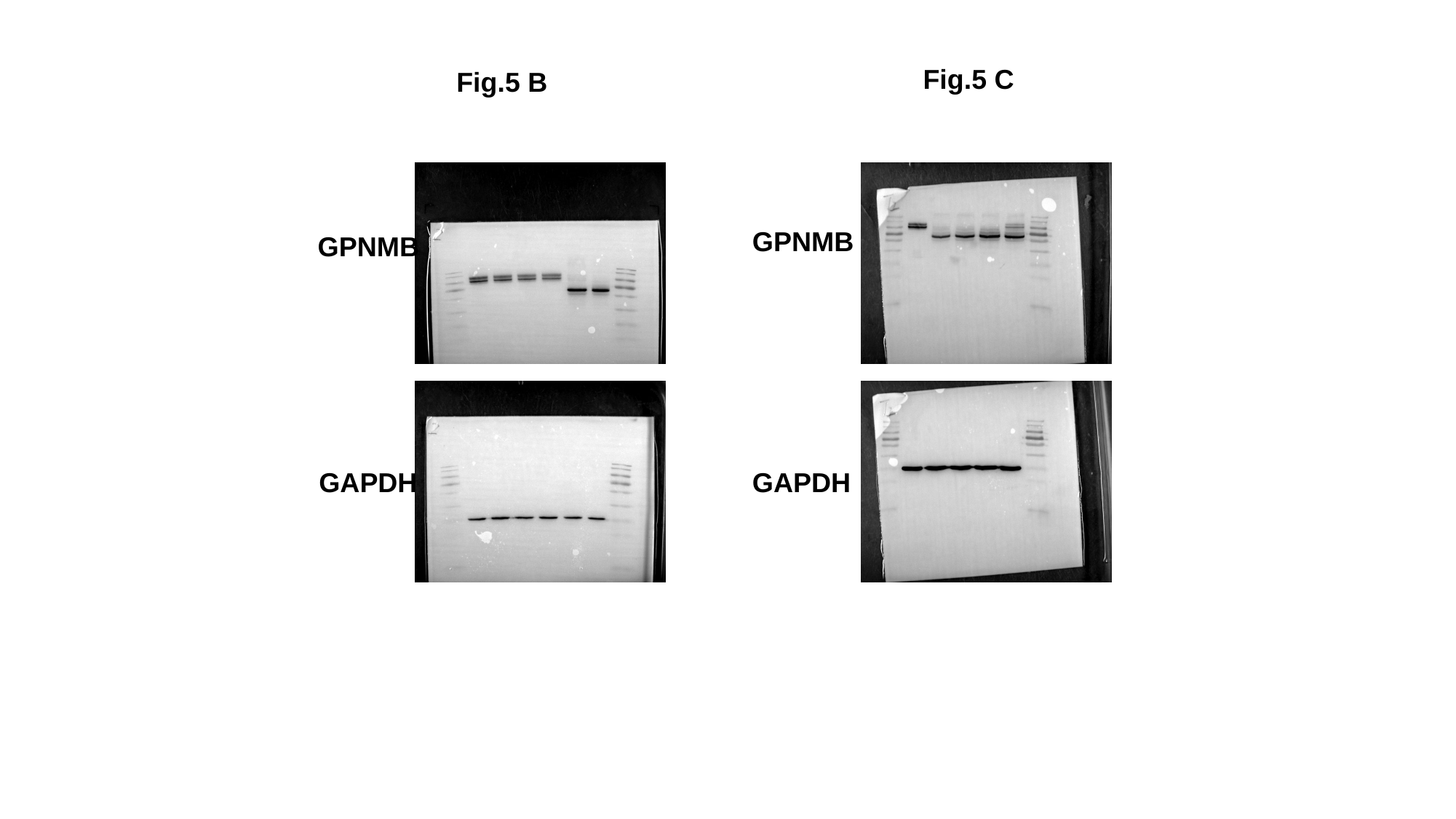

Fig.5 C
Fig.5 B
GPNMB
GPNMB
GAPDH
GAPDH

## Slide 4
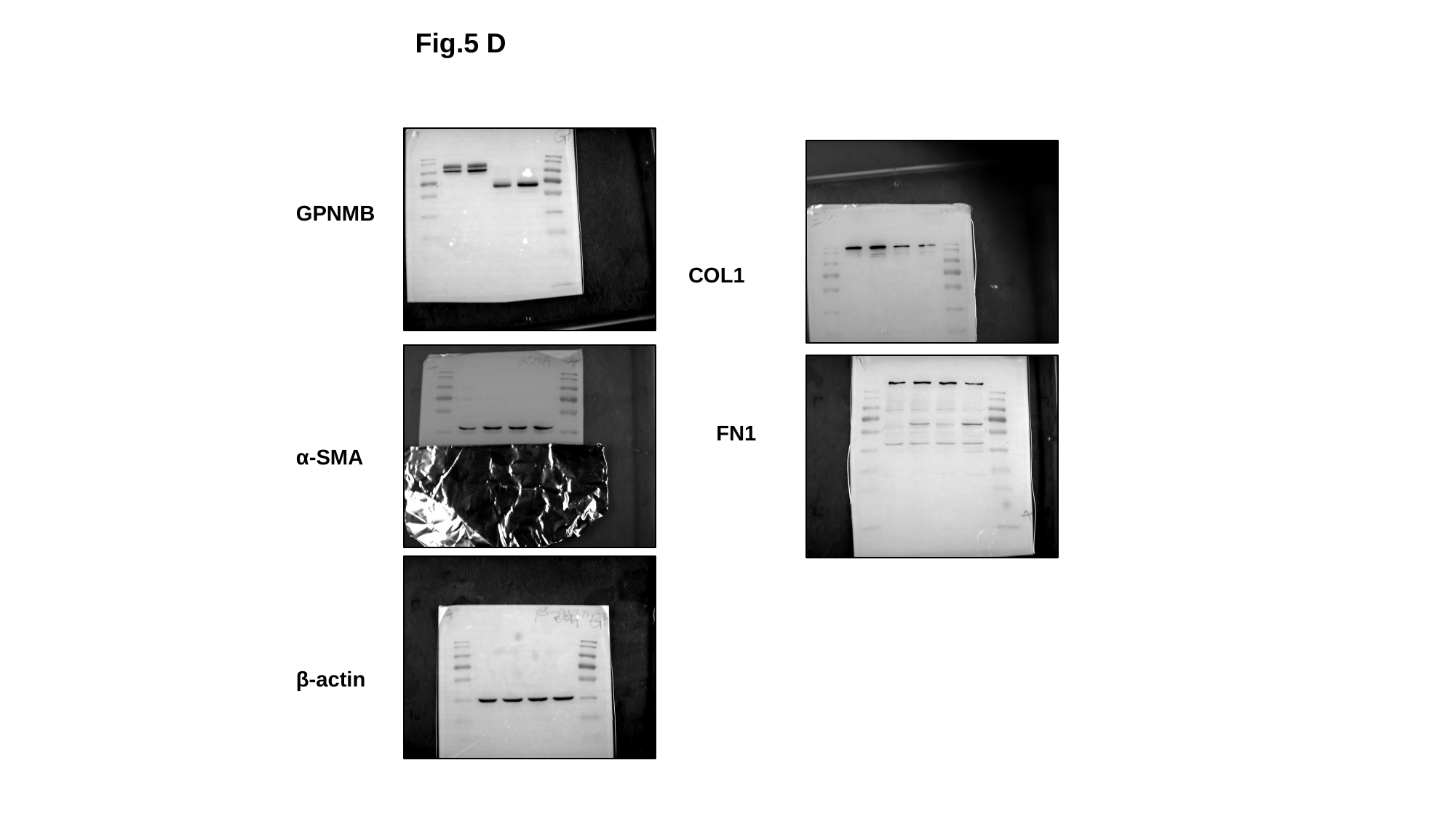

Fig.5 D
GPNMB
COL1
FN1
α-SMA
β-actin

## Slide 5
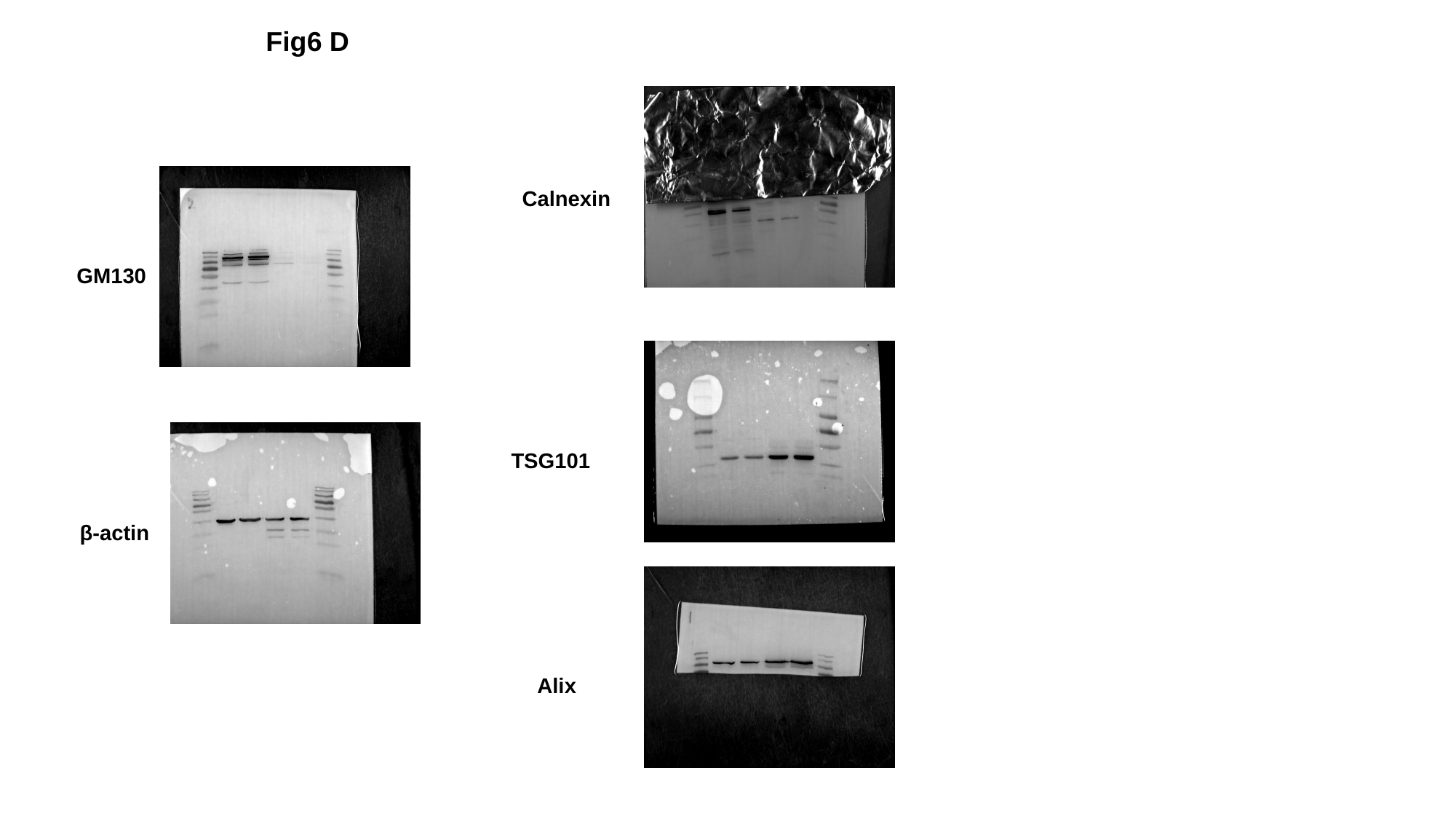

Fig6 D
Calnexin
GM130
TSG101
β-actin
Alix

## Slide 6
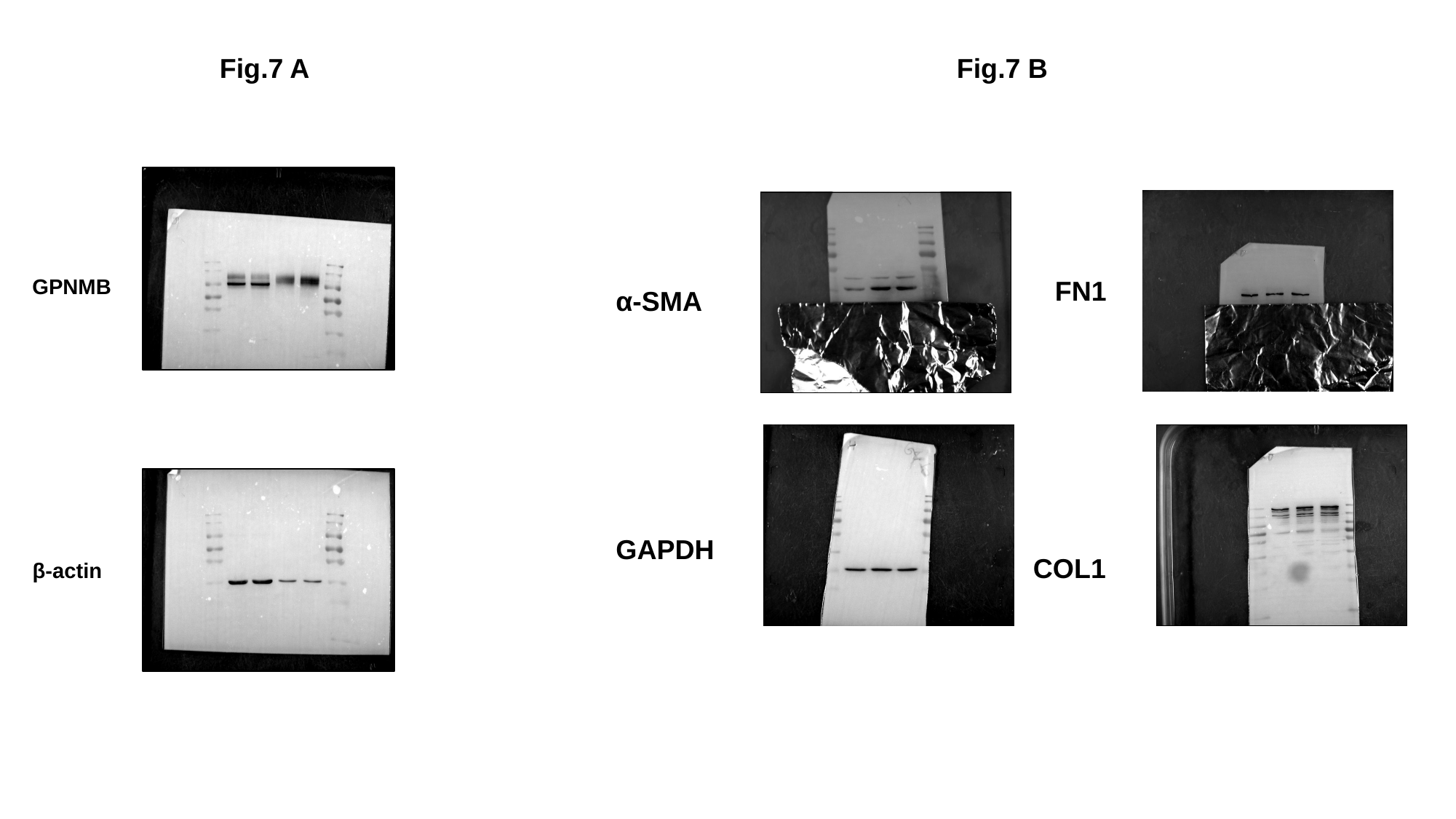

Fig.7 A
Fig.7 B
GPNMB
FN1
α-SMA
GAPDH
COL1
β-actin

## Slide 7
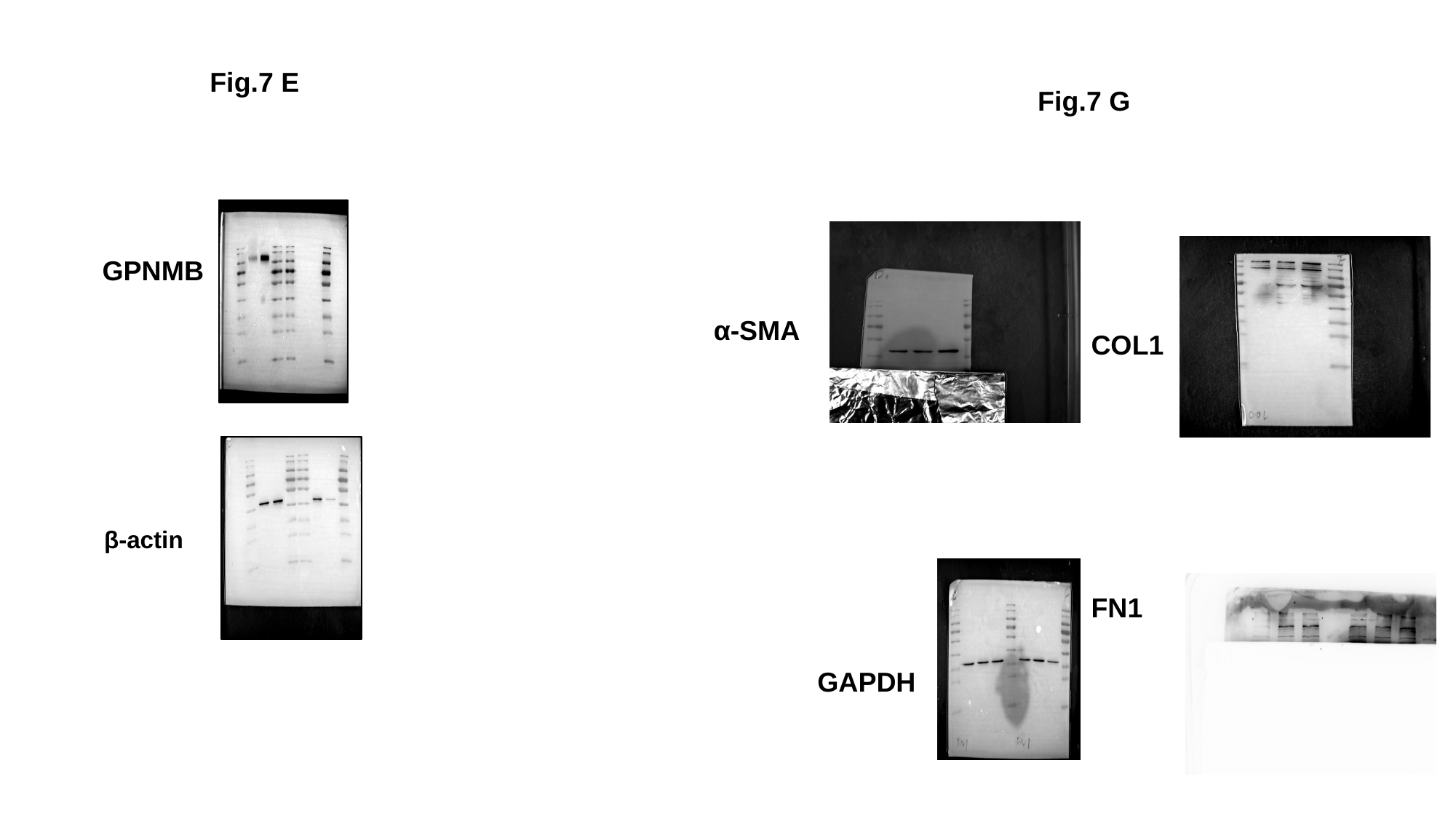

Fig.7 E
Fig.7 G
GPNMB
α-SMA
COL1
β-actin
FN1
GAPDH
