# Supplementary material for: Single-cell transcriptome sequencing–based analysis: probing the mechanisms of glycoprotein NMB regulation of epithelial cells involved in silicosis
Source: Part Fibre Toxicol. 2023 Jul 19;20:29. doi: 10.1186/s12989-023-00543-9 (PMC10354944; doi:10.1186/s12989-023-00543-9)
Supplement: Supplementary file 2 — Supplementary Material 2 [file 12989_2023_543_MOESM2_ESM.docx]

**Supplementary Figure 1.**

**
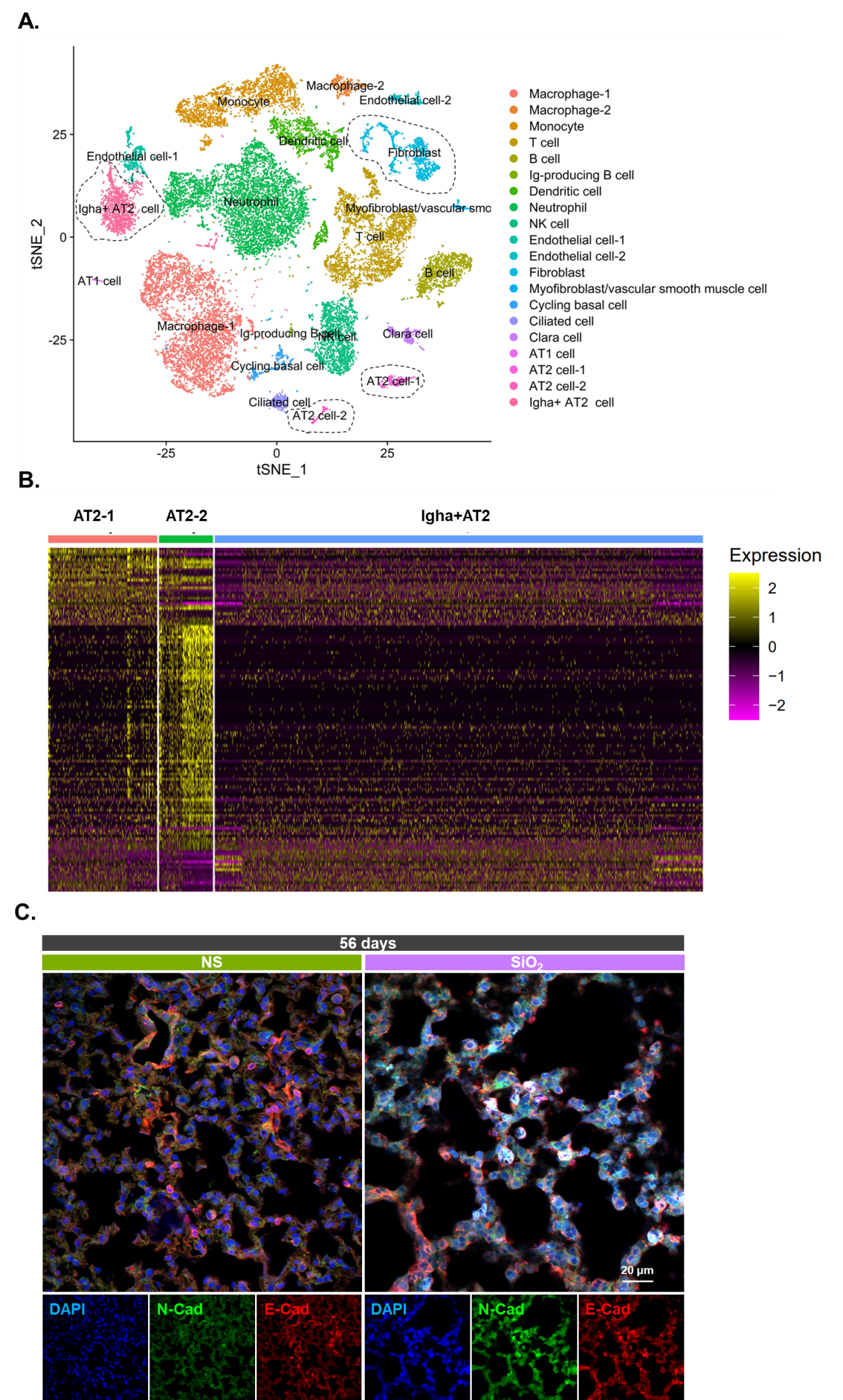
**

**Supplementary Figure 1. Fractionation basis of alveolar type II epithelial cells**

A. scRNA-Seq clustering and definitions. The cell type for each cluster was annotated according to canonical cell markers in CellMarker.

B. Heat map representing the expression of distinct RNAs that identify three groups of AT2 cell subtypes.

C. Immunohistochemical staining showing that E-Cad and N-Cad were expressed in lung tissue, scale bar: 20 μm.

**Supplementary Figure 2.**

**
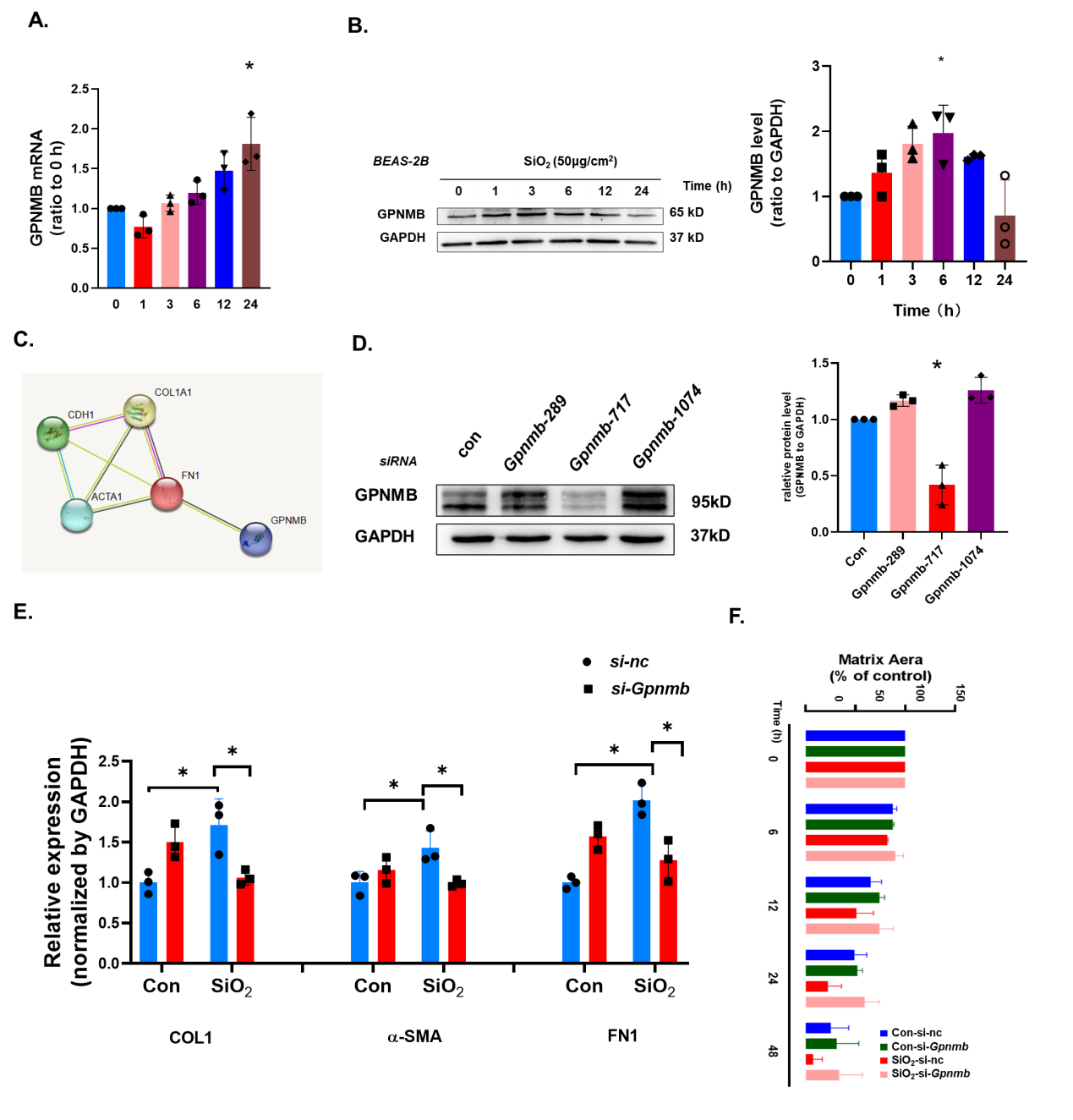
**

**Supplementary Figure 2. Experimental evidence of the EMT-related phenomena of MLE-12 cells**

A. GPNMB expression was elevated over time in MLE-12 cells, with significant differences in expression between 24 h and 0 h (**P* < 0.05).

B. Representative western blot results showing that the elevated GPNMB expression in alveolar type II epithelial cells can activate EMT through the upregulation of GPNMB cells; #*P* < 0.05 indicates significant differences in expression between 6 h and 0 h (**P* < 0.05).

C. Interaction map showing interactions between proteins enriched in GPNMB and interstitial marker protein.

D. Western blot showing that the knockdown efficiency of *Gpnmb*-177 using one-way ANOVA suggested a statistically significant difference.

D. **P* < 0.05 indicates that the SiO_2_-treated *si-nc* group had higher protein levels than the control group and that the model was successfully established; #*P* < 0.05 indicates that *si-Gpnmb* reversed the SiO_2_-induced elevated protein levels of COL1, α-SMA, and FN1 after SiO_2_ treatment.

E. **P* < 0.05 indicates that the cell migration rate was higher in the *si-nc* group than in the control group after SiO_2_ treatment and that the model was successfully established; #*P* < 0.05 indicates that the cell migration rate was lower in the *si-Gpnmb* group than in the *si-nc* group after SiO_2_ treatment.

F. **P* < 0.05 indicates that the cell migration rate in the *si-nc* group was higher than that in the control group after SiO_2_ treatment and that the model was successfully established; #*P* < 0.05 indicates that the cell migration rate was lower in the *si-Gpnmb* group than in the *si-nc* group after SiO_2_ treatment.

**Supplementary Figure 3.**

**
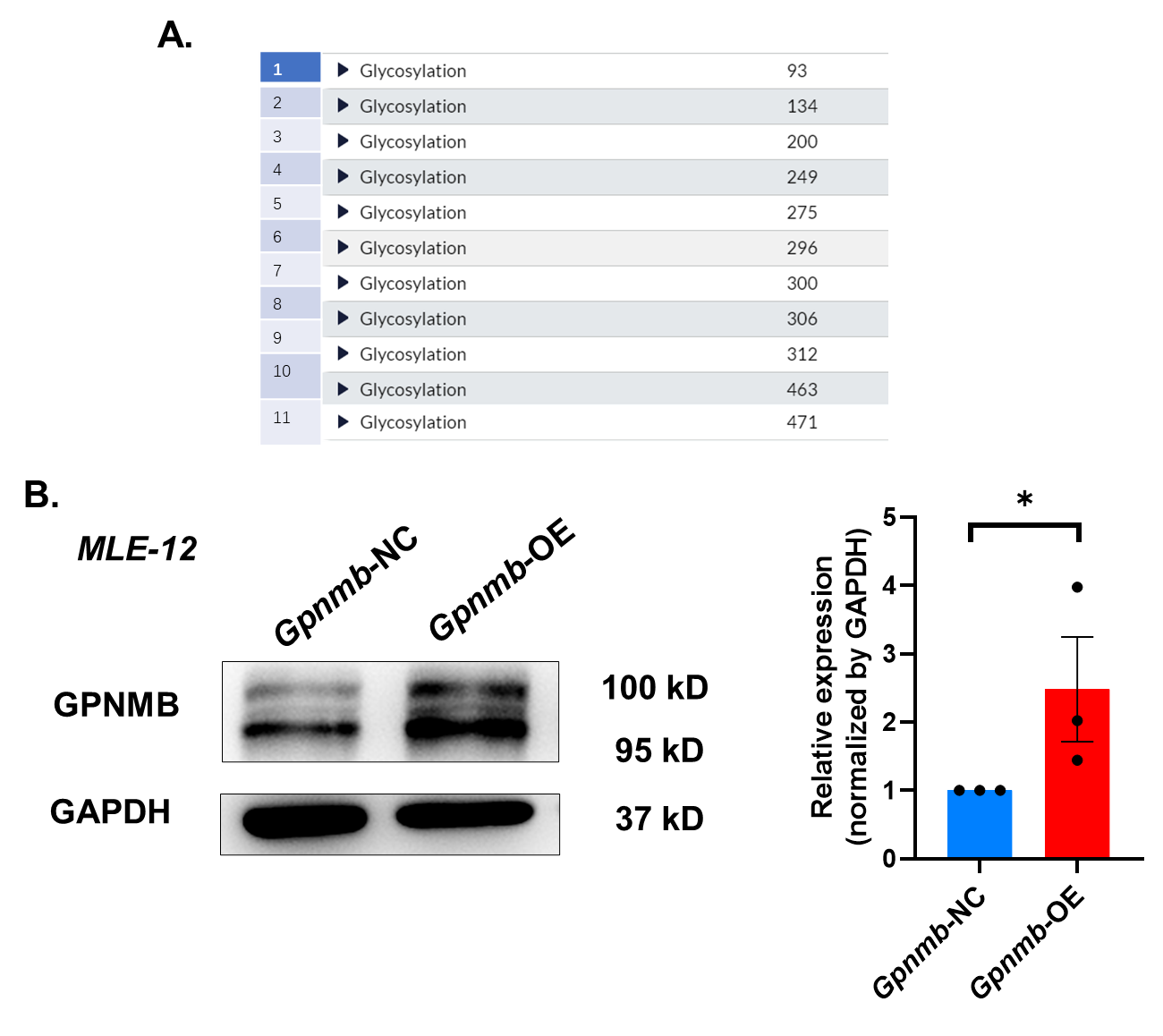
**

**Supplementary Figure 3. The glycosylation level of GPNMB**

A. Eleven GPNMB glycosylation sites.

B. Western blot showing the successful establishment of *Gpnmb* overexpressing cell lines; **P* < 0.05 indicates statistically significant differences.

**Supplementary Figure 4.**

**
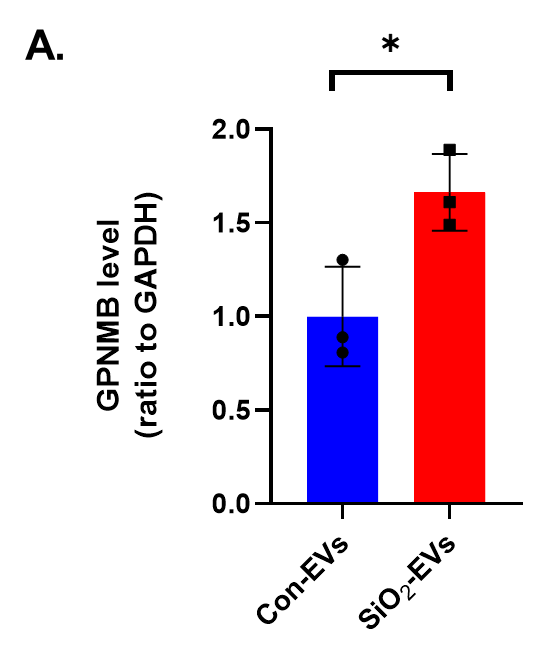
**

**Supplementary Figure 4. Identification of GPNMB expression in EVs**

**P* < 0.05 indicates that the level of GPNMB protein was higher in the SiO_2_ group than in the control group.

**Supplementary Figure 5.**

**
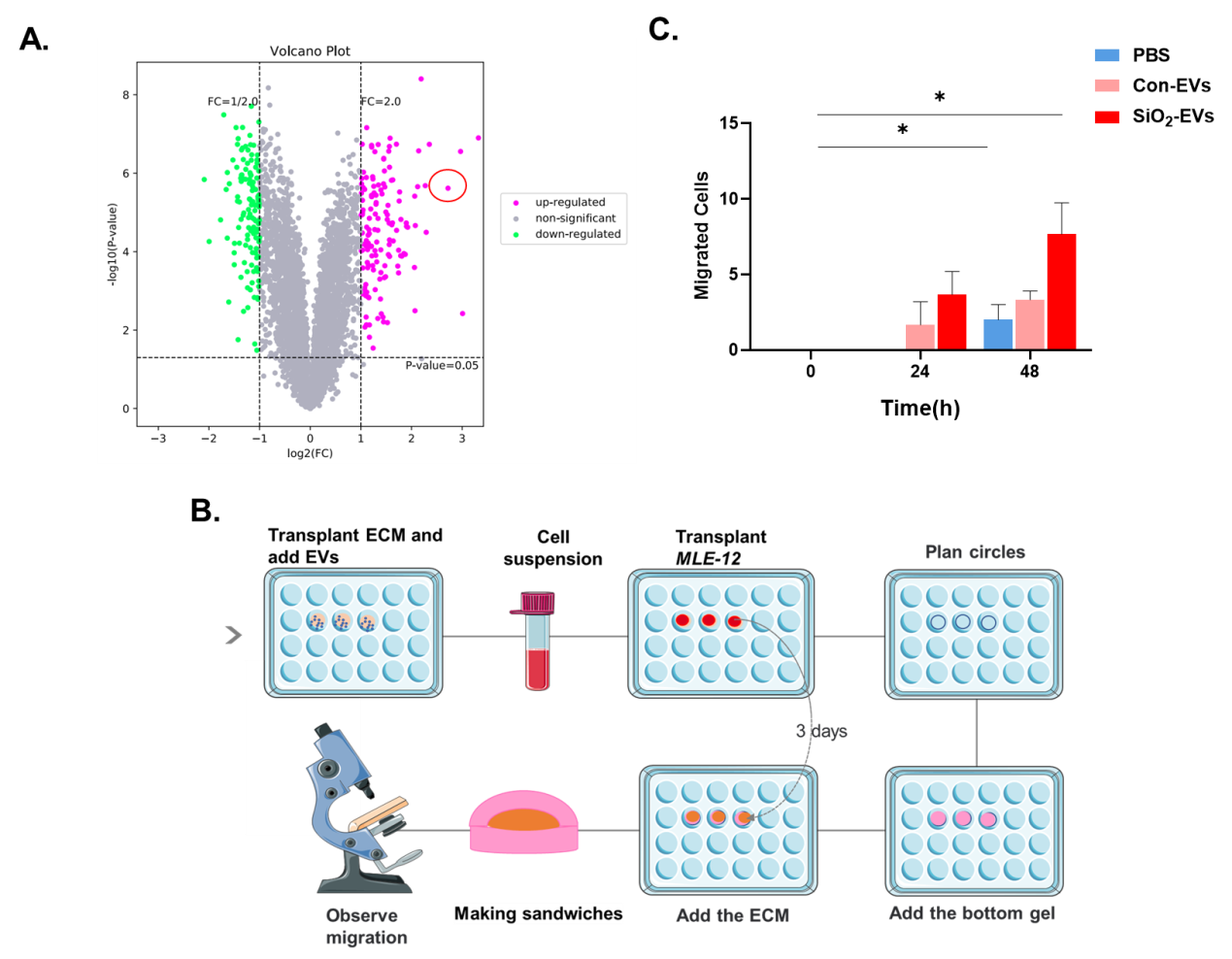
**

**Supplementary Figure 5. Process of EVs adhesion to the ECM.**

A. According to the ECM proteomics results, 270 proteins showed differential expression in the ECM derived from the lung tissue of silicosis mice.

B. Schematic diagram of the nested matrix model method.

C. Number of cells migrating at different time points; **P* < 0.05 shows a statistically significant difference at 48 h compared to 0 h.
